# Effects of Pressure, Temperature, Treatment Time, and Storage on Rheological, Textural, and Structural Properties of Heat-Induced Chickpea Gels

**DOI:** 10.3390/foods4020080

**Published:** 2015-04-15

**Authors:** María Dolores Alvarez, Raúl Fuentes, Wenceslao Canet

**Affiliations:** Department of Characterization, Quality, and Safety, Institute of Food Science, Technology, and Nutrition (ICTAN-CSIC), José Antonio Novais 10, 28040 Madrid, Spain; E-Mails: raul.fuentes@ictan.csic.es (R.F.); wenceslao@ictan.csic.es (W.C.)

**Keywords:** chickpea flour, high hydrostatic pressure, oscillation rheology, texture, gelatinization, retrogradation, structure

## Abstract

Pressure-induced gelatinization of chickpea flour (CF) was studied in combination with subsequent temperature-induced gelatinization. CF slurries (with 1:5 flour-to-water ratio) and CF in powder form were treated with high hydrostatic pressure (HHP), temperature (*T*), and treatment time (*t*) at three levels (200, 400, 600 MPa; 10, 25, 50 °C; 5, 15, 25 min). In order to investigate the effect of storage (*S*), half of the HHP-treated CF slurries were immediately analyzed for changes in oscillatory rheological properties under isothermal heating at 75 °C for 15 min followed by cooling to 25 °C. The other half of the HHP-treated CF slurries were refrigerated (at 4 °C) for one week and subsequently analyzed for changes in oscillatory properties under the same heating conditions as the unrefrigerated samples. HHP-treated CF in powder form was analyzed for changes in textural properties of heat-induced CF gels under isothermal heating at 90 °C for 5 min and subsequent cooling to 25 °C. Structural changes during gelatinization were investigated using microscopy. Pressure had a more significant effect on rheological and textural properties, followed by *T* and treatment *t* (in that order). Gel aging in HHP-treated CF slurries during storage was supported by rheological measurements.

## 1. Introduction

Chickpea (*Cicer arietinum* L.) is a legume that is very commonly used in many countries because of its ideal cell wall polysaccharide composition and starch properties [1]. According to the authors just cited, although legumes contain relevant levels of proteins, carbohydrates, dietary fiber, and vitamins, they also present bioactive substances including enzyme inhibitors, lectins, phytates, phenolic compounds, fatty acids, bioactive peptides, and oligosaccharides (raffinose family of oligosaccharides), which have been reported potential health benefits. Foods based on this legume are prepared using a wide range of recipes and preparation procedures, among which heat processing is a well-established method. Chickpea flour (CF) slurry can form a gel under suitable processing conditions, and the gelling ability of chickpea flour/starch and the viscous nature of the cooked paste are important for the manufacture and development of chickpea-based food gels. In starch-containing foods, the structure of a gel/paste is dictated by several factors [2].

In order to find ideal processing characteristics, two or more processing methods are commonly applied simultaneously [3]. HHP is a promising non-thermal technology for the development of fresh-like, shelf-stable foods. In general, heat and pressure have similar effects: if sufficiently high, they both induce gelatinization of starch in excess water, resulting in a transition of the native granular structure to a starch paste or gel [4]. However, there are significant differences in the structural and rheological properties of heated and pressurized starches. These differences offer benefits with respect to new product development.

Therefore, improved use of chickpea can be obtained by implementing various processing strategies to facilitate development of easily manageable alternative products with optimized sensory qualities [5]. The authors just cited investigated the thermorheological changes in HHP-treated CF slurries as a function of pressure level (0.1, 150, 300, 400, and 600 MPa) and slurry concentration (1:5, 1:4, 1:3, and 1:2 flour-to-water ratios). HHP-treated slurries were analyzed for changes in properties produced by heating, under both isothermal and non-isothermal processes. CF slurries subjected to 600 MPa at 25 °C for 15 min showed no peak and hence no enthalpy value, suggesting complete HHP-induced gelatinization of starch. Heat-induced CF paste gradually transformed from solid-like behavior to liquid-like behavior as a function of concentration and pressure level. In terms of industrial application, it has been observed that raw gel/paste formed by heating of chickpeas in water is very hard if the objective is a paste that is to be flattened/rolled/sheeted/shaped to make different products. The addition of HHP-treated CF slurries (600 MPa at 25 °C for 15 min) to similar unpressurized batter-based products would offer easier flow characteristics during heating, preparation, and handling [5]. 

However, it has been shown that pressure-induced starch gelatinization is highly sensitive to changes in pressure, *T*, and treatment *t*. The applicability of HHP-induced starch gelatinization as a pressure–time–temperature indicator was investigated by examining the impact of pressure, *T*, and treatment *t* on different starches [6], and different combinations of the three factors could result in the same degree of gelatinization [7]. The authors just cited studied the effects of treatment pressure (≤700 MPa), treatment *T* (10–60 °C), and treatment duration (0–30 min) on the gelatinization of normal and waxy rice starches, and a linear correlation between the degree of swelling and the initial apparent viscosity was observed. The same degree of gelatinization can be brought about by different pressure–*t* combinations, *i.e.*, lower pressure can, to a certain extent, be compensated for by longer treatment *t* [8]. When sorghum starch suspensions were treated at increasing pressure (200–600 MPa) or *T* (60–95 °C) for 10 min, both pressure- and *T*-induced gelatinization curves were sigmoid-shaped [9]. On the other hand, HHP-treated CF slurries were also studied as a function of pressure level at fixed *T* and treatment *t* (0.1, 150, 300, 450, and 600 MPa at 25 °C for 15 min) and slurry concentration (1:5, 1:4, 1:3, and 1:2 flour-to-water ratios), and elastic modulus (*G*’) increased with pressure applied and concentration indicating that the degree of gelatinization increased with increasing pressure [5]. However, although the same degree of gelatinization can be brought about by different pressure–*T*–*t* combinations, the starch properties of the CF slurries such as pasting behavior, degree of swelling, hydration of the starch, and changes in birefringence can be significantly very different. The HHP dependence of wheat and corn starches cannot be determined via the traditional microscopical method, but needs the application of the differential scanning calorimetry (DSC) investigation, which became the preferred method for the determination of the gelatinization pattern [10]. DSC determines the difference in the amount of heat required to increase the temperature of a sample and reference as a function of temperature [9]. The authors just cited observed that in sorghum starch-water suspensions the melting enthalpy decreased with increasing pressure or temperature, and, consequently, the percentage of gelatinized starch granules increased. The decrease of the gelatinization enthalpy with increasing pressure was also reported for different aqueous starch suspensions [10] and CF slurries [5]. 

DSC has also been used to study starch retrogradation phenomena during HHP processing [8,11,12,13]. The retrogradation of pressure-induced gels is thought to differ from that of heat-induced gels because almost no leaching of amylose is observed after pressurization, and therefore the retrogradation occurs within the starch granules [13]. However, the extent of the pressure effect on the crystalline structure depends on the type of crystallinity [4]. A higher resistance of B-type starches to pressure has been reported by Stute *et al*. [10]. The sensitivity of the C-type starches is located in between B- and A-type starches: Bauer and Knorr [6] showed that the phase gelatinization of tapioca starch (C-type) occurred at higher pressures than the phase change of wheat starch (A-type) but below the melting of potato starch. Pressure-treated lentil samples exhibited a relatively lower extent of recrystallization than thermally-treated slurry during storage [12]. For retrogradation studies, a 25% starch suspension was pressurized at 550 MPa and 30 °C for 10 min. This treatment induced a gel for which no endothermic peak was observed in the DSC thermogram immediately after pressurization [8]. However, after one day of storage at 4 °C, a small, broad peak—typical for retrogradation—appeared, and the enthalpy of the amylopectin crystals formed during storage increased with increasing storage time. In turn, Ahmed *et al*. [11] reported that there was no retrogradation in HHP-treated starch samples during low temperature storage for 24 h. In the present work, half of the samples were analyzed immediately after HHP treatment, whereas the other half were stored for one week at 4 °C prior to *T*-treatment and subsequent analysis.

The effect of high pressure on starch has been explored by many researchers using a wide range of techniques [4]. The investigations performed thus far on the effect of high pressure on starch can be divided into three categories [10]. First are those in which the pressure applied was not high enough to provide a gelatinization effect [14]. Second are those in which the pressure application was performed on almost dry starch [15,16]. Third are those where the investigations were carried out with excess water and pressures above 400 MPa [4,5,9,10]. This study focused on the three categories, combining gelatinization induced by HHP treatment with the better understood thermal gelatinization. However, this categorization should be viewed with caution. If the pressure was not high enough to provide effects, this could be due to the type of starch or due to the excess water content in the suspension. A pressure above 400 MPa at excess water is, e.g., not sufficient for potato starch gelatinization but shows effects for wheat starch [10]. In other cases, the pressure was high enough but the water content was too low. All the factors such as HHP, *T* at pressurization, treatment *t*, starch type, and other major components (protein, fat, ash, salts, *etc.*) interact and contribute to the effect of pressure-induced gelatinization of starch.

Little information is available concerning the impact of HHP on whole flour systems. In addition to starch, these CF slurries contain a relatively large amount of protein (20.64 ± 0.05 g 100 g^−1^), which could give a second endothermic peak on heating in water [5]. Ahmed *et al*. [11] studied the effect of HHP treatment of basmati rice flour slurries and found both gelatinization of starch and denaturation of proteins. In turn, Ahmed *et al*. [12] studied the thermal characteristics of HHP-treated lentil flour slurries at selected moisture levels and found no starch gelatinization peak during thermal scanning. In contrast, the authors just cited provided complementary information of HHP-induced structural changes on both the molecular and the sub-molecular level of lentil protein.

The objective of this work was to evaluate the combined effects of treatment pressure (200, 400, and 600 MPa), *T* at pressurization (10, 25, and 50 °C), treatment *t* (5, 15, and 25 min), and storage (*S*) on the subsequent thermal gelatinization of HHP-treated CF slurry and HHP-treated CF in powder form, with a view to providing information that can be used to develop CF-based food products with advantageous handling properties. For this purpose, unpressurized and HHP-treated CF slurries were then pasted under isothermal heating conditions at 75 °C for 15 min, cooled to 25 °C, and then evaluated for changes in their dynamic gel and structural properties. In turn, unpressurized and HHP-treated CF in powder form was pasted at 90 °C for 5 min, cooled to 25 °C, and subsequently evaluated for changes in its textural and structural properties.

## 2. Experimental Section

### 2.1. Materials

Spanish chickpea (*C. arietinum* cv. Castellano) flour was a commercially available product donated by the Los Pisones flour milling company (Zamora, Spain). CF was supplied packed in polyethylene pouches (500 g) and was stored in watertight containers (10 °C and 73% ± 3% relative humidity) until use. Mean values for proximate analysis (g 100 g^−1^) of CF samples, as analyzed by the AOAC method [17], were: moisture, 8.49 ± 0.34, total ash, 2.77 ± 0.24, and crude protein (N × 6.25), 20.64 ± 0.05.

### 2.2. CF Slurry Preparation

CF slurries were prepared at a concentration of 1:5 flour-to-water ratio. The required amounts of CF and distilled water were placed in a 250-mL beaker, hand-mixed with a glass rod, and kept for half an hour at room *T* (25 ± 1 °C) for hydration with stirring at 900 rpm before subjecting each sample to the HHP treatments.

### 2.3. High Hydrostatic Pressure Treatment

**Figure 1:**
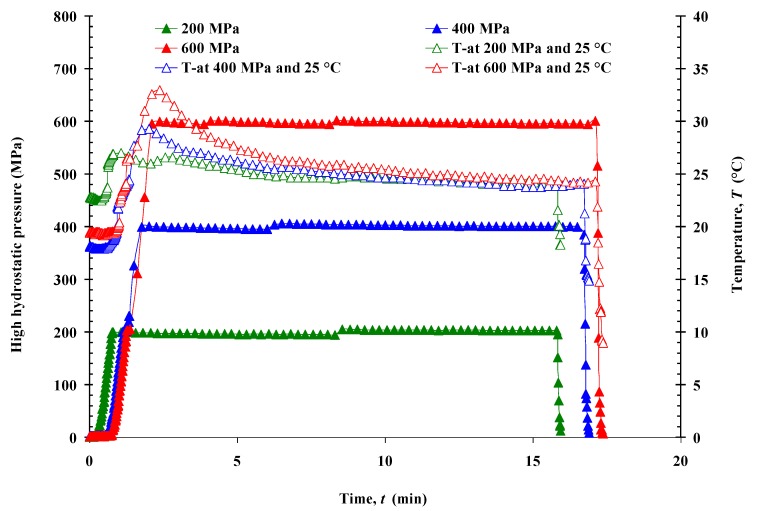
Representation of the temperature and pressure *vs.* time variation during HHP treatments (200, 400, and 600 MPa at 25 °C for 15 min).

Either CF slurry (200 mL) or CF in powdered form (200 g) was vacuum packaged in a very low gas permeability bag type, Doypack^®^ (Polyskin XL, Flexibles Hispania, S.L., Spain). Packed samples were vacuum-packed one more time to prevent contact between pressurization fluid and slurry. HHP treatment was performed using a Stansted Fluid Power Iso-lab 900 High Pressure Food Processor (Model: FPG7100:9/2C, Stansted Fluid Power Ltd., Harlow, Essex, UK), with 2925 mL capacity, maximum pressure of 900 MPa, and a potential maximum temperature of 100 °C. Four packed samples were inserted simultaneously into the pressure unit filled with pressure medium (water), then treated at a pressure of 200, 400, or 600 MPa, and compared with an untreated sample. The pressure was increased at a rate of 500 MPa min^−1^ and maintained at the desired level for holding *t*s of 5, 15, and 25 min; the decompression *t* was less than 4 s. The *T* of the pressure unit vessel was thermostatically controlled at 10, 25, and 50 °C throughout the different treatment combinations. Pressure, *T*, and *t* were controlled by a computer program, being constantly monitored and recorded during the process. Representations of the variation of *T* *vs.* *t* during HHP treatments carried out at 25 °C for 15 min (at 200, 400, and 600 MPa) are shown in Figure 1. Increases of up to a maximum of 8 ± 1 °C, 13 ± 2 °C, or 17 ± 2 °C at 200, 400, or 600 MPa, respectively, due to compressive heating, were observed in the *T* of the pressurizing fluid, but they were transient and equilibrated at 10, 25, and 50 ± 2.5 °C during the holding period at those pressure levels. The average adiabatic heating during pressurization was ~3.4 °C/100 MPa. After HHP treatment, half of the samples were immediately analyzed, and the other half were stored in a refrigerator at 4 °C for one week before the actual measurements in order to investigate the retrogradation behavior of stored samples in the aging process. All the HHP treatments were performed twice (two batches).

### 2.4. Rheological Measurements

Rheological measurements were carried out on freshly HHP-treated CF slurry and after 1 week of storage at 4 °C. A Kinexus pro rotational rheometer (Malvern Instruments Ltd., Worcestershire, UK) was used to make SAOS measurements under non-isothermal heating conditions and at 25 °C in combination with a concentric cylinder geometry (C25 DIN SW1009 SS: PC25 DIN CO155 AL), and a solvent trap was used to minimize moisture loss during tests. The sample *T* was internally controlled by a Peltier system (−40 to 200 °C with an accuracy of ±0.1 °C) attached to a water circulation unit. For each test, a measured volume of thoroughly mixed sample (approximately 18 mL) was placed in the outer cylinder, PC25. CF slurry was pasted under isothermal heating conditions in the concentric cylinder geometry *in situ* maintained at 75 °C, using the pre-condition option for 15 min (time sweeps), with controlled amplitude of the periodic shear stress (*σ*) at a constant value of 1 Pa to guarantee the existence of linear viscoelastic (LVE) response, and at a frequency (*f*) of 1 Hz. After pasting, the sample was immediately cooled down to 25 °C at 2 °C min^–1^; *f* and *σ* signals were maintained at constant values of 1 Hz and 1 Pa, respectively. In order to ensure that all measurements were carried out within the LVE range, initially oscillation stress amplitude sweeps at 25 °C were tested at 1 Hz by varying the *σ* of the input signal from low (0.01, 0.1, or 1 Pa) to high levels, depending on the HHP treatment. Mechanical spectra in the linear region at variable frequency (*ω*) from 1 to 100 rad s^−1^ (~0.16 to 16 Hz) were also recorded in separate tests. Elastic and viscous moduli (*G*’ and *G*”, respectively), complex viscosity (η*, Pa s), and loss tangent (tan *δ* = *G*”/*G*’, dimensionless) values at a *f* of 1 Hz (~6.28 rad s^−1^) were chosen to compare gel rigidity.

Given that the appearance of the data on logarithmic coordinates was nearly linear at 25 °C, a power law model was used to characterize *f* dependence of the elastic and viscous moduli as follows (Equations (1) and (2)):

*G*’ = *G*’_0_*ω^n^*^’^ (1)

*G*” = *G*”_0_*ω^n^*^”^ (2)

where *G*’_0_ (Pa s*^n^*^’^), and *G*”_0_ (Pa s*^n^*^”^) are elastic and viscous moduli at 1 Hz, respectively, and exponents *n*’ and *n*” (both dimensionless) denote the influence of *f* on the two moduli [18]. The difference (*G*’_0_ − *G*”_0_) was also used as a measure of *gel strength* [19]. All rheological measurements were carried out in triplicate for each batch (six replicates were measured), and rheological properties were obtained directly from the manufacturer-supplied computer software (rSpace for Kinexus v. 1.40, Malvern Instruments Ltd., Worcester, UK).

### 2.5. Chickpea Gel Preparation from HHP-Treated Flour in Powder Form

Chickpea gels were prepared in 562 g batches from 80 g of unpressurized or HHP-treated flour, 480 mL of water, and 2 g of salt (NaCl). A TM 31 food processor (Vorwerk España, M.S.L., S.C., Madrid, Spain) was used to prepare the chickpea gels. In the food processor, the flour and the salt were dispersed in the water, and the ingredients were first allowed to reach 90 °C and then cooked for 5 min at this temperature, stirring constantly at 300 rpm (speed 2). Then the dough was immediately transferred to a stainless steel container and cooled by employing a cold water bath with ice for 1 h prior to any further measurement in order to set the gel faster [20].

### 2.6. Instrumental Texture Measurements

The texture measurements of CF gel heat-induced from either unpressurized or HHP-treated flour were carried out with a TA.HDPlus Texture Analyzer (Stable Micro Systems Ltd., Godalming, UK) equipped with a 49 N load cell. Two different tests were performed: a bloom strength (BS) test and a texture profile analysis (TPA). During all the tests, the chickpea samples were maintained at 25 °C by means of a temperature-controlled Peltier cabinet coupled to a separate heat exchanger and proportional-integral-derivative control unit. Texture measurements were carried out 10 times for each batch (20 replicates were measured).

#### 2.6.1. BS Test

In this test, the gels were penetrated using the standardized cylindrical probe (P/O.5, Stable Micro Systems Ltd., Godalming, UK) for gelatin bloom testing with a diameter of 12.7 mm and no curvature radius in accordance with ISO 9665:1998(E) [21]. The gels were penetrated up to a distance of 15 mm, using a trigger force of 0.04 N and a test speed of 0.5 mm s^−1^. Instrumental parameters obtained from the force–distance curves were: the bloom strength as the weight needed by the probe to deflect the surface of the gel 4 mm without breaking, *BS* (g), the rupture strength as the maximum force, *RS* (N), the adhesiveness as the negative area during the probe reversal, *Adh* (*BS*) (N s), and the brittleness as the slope at maximum force, *Slope* (N mm^−1^) [20].

#### 2.6.2. TPA Test

Cylindrical specimens with a diameter of 25.4 mm and height of 10 mm were cut from the chickpea gels using a stainless steel cork borer and a mechanically guided razor blade specially designed for this diameter [22]. They were double-compressed using the flat 45-mm-diameter aluminum plunger (SMS, P/45) up to a distance of 7 mm, with a rest period of 2 s between cycles. The trigger force was 0.05 N and the test speed was 5 mm s^−1^. The force–time curves gave the hardness, *Hard* (N), adhesiveness, *Adh* (N s), springiness, *Spr* (dimensionless), and cohesiveness, *Coh* (dimensionless). Definitions of textural parameters are given elsewhere [23].

### 2.7. Scanning Electron Microscopy (SEM)

The microstructure of CF gels heat-induced from unpressurized and HHP-treated CF slurries, and of unpressurized and HHP-treated CF in powder form was examined by using a Hitachi S-2100 scanning electron microscope (Hitachi, Ltd., Tokyo, Japan) (National Center for Metallurgical Research (CENIM)-CSIC). Samples were air-dried, then mounted and sputter-coated with Au (200 A approx.) in an SPI diode sputtering system metalizer. Micrographs were taken with a Scanvision 1.2 digital system (Röntgenanalysen-Technik (RONTEC) GmbH, Berlin, Germany) (800 × 1200 pixels).

### 2.8. Statistical Analysis

To examine the impact of pressure, *T*, treatment *t*, and storage (*S*) on the rheological properties of heat-induced CF gels under isothermal heating at 75 °C for 15 min and after subsequent cooling to 25 °C, a four-way analysis of variance (ANOVA) with interactions was used, including the unpressurized sample. Additionally, to establish the effect of pressure at constant *T* and treatment *t*, the effect of treatment *T* at constant pressure and treatment *t*, and the effect of treatment *t* at constant pressure and *T*, on samples without and with *S*, six one-factor ANOVAs were performed separately for the different combinations of factors. In addition, to evaluate the effect of *S* at constant pressure, *T*, and treatment *t*, 28 one-factor ANOVAs were carried out independently. In turn, to study the effect of pressure, *T*, and treatment t on the textural properties of CF gels heat-induced from pressurized powder form, a three-way ANOVA with interactions was used, including the unpressurized sample. Furthermore, to establish the effect of pressure at constant *T* and treatment *t*, the effect of treatment *T* at constant pressure and treatment *t*, and the effect of treatment *t* at constant pressure and *T*, three one-factor ANOVAs were performed separately for the different combinations of factors. The mean values shown were obtained from two batches of HHP-treated samples, and three replicates were measured for each batch. Minimum significant differences were calculated by Fisher’s least significant difference (LSD) multiple range test at a significance level of 0.05. Statistical analyses were carried out using the SPSS 19.0 statistical software package (SPSS, Inc., Chicago, IL, USA).

## 3. Results and Discussion

### 3.1. Effect of Pressure, Temperature (T), Treatment Time (t), and Storage (S) on Rheological Properties of Heat-Induced CF Gel

The four-way mixed-model ANOVA showed significant (*p* < 0.05) pressure and treatment *T* main effects for all the rheological properties derived from mechanical spectra (Table 1). Treatment *t* and storage *S* were not significant for *n*” and *n*’ values (regression coefficient relating *G*” or *G*’ and *f*), respectively. Even so, pressure had a more significant effect on rheological parameters (higher *F* values), followed by *T*, treatment *t*, and *S* (in that order). In addition, pressure, *T,* treatment *t*, and *S* had a more significant effect on the loss modulus (*G*”) values (higher *F* values).

**Table 1:** Four-way analysis of variance of the rheological properties of heat-induced chickpea flour gel at 75 °C for 15 min derived from mechanical spectra after cooling to 25 °C (high hydrostatic pressure treatments: 200, 400, and 600 MPa; 10, 25, and 50 °C; 5, 15, and 25 min, in samples without and with storage, and unpressurized slurry). *F* and *p* values.

Parameter	Pressure (*df* = 2)				Temperature (*T*) (*df* = 2)				Time (*t*) (*df* = 2)				Storage (*S*) (*df* = 1)			Pressure × *T* (*df* = 4)				Pressure × *t* (*df* = 4)				Pressure × *S* (*df* = 2)			*T* × *t* (*df* = 4)		
	*F* value		*p* value		*F* value		*p* value		*F* value		*p* value		*F* value	*p* value		*F* value		*p* value		*F* value		*p* value		*F* value		*p* value	*F* value		*p* value
*G*’(Pa)	3765.9		0.000		1144.7		0.000		616.0		0.000		9.8	0.002		365.5		0.000		29.8		0.000		1743.6		0.000	544.3		0.000
*G*” (Pa)	8883.9		0.000		2083.6		0.000		1308.3		0.000		63.8	0.000		430.5		0.000		29.4		0.000		3678.7		0.000	1282.7		0.000
tan *δ* (-)	566.5		0.000		255.6		0.000		61.4		0.000		6.4	0.013		148.9		0.000		18.3		0.000		13.2		0.000	6.6		0.000
*n*’(-)	75.4		0.000		20.7		0.000		5.8		0.004		1.9	0.173		35.0		0.000		1.0		0.428		0.15		0.861	1.2		0.320
*n”* (-)	21.0		0.000		14.7		0.000		2.8		0.064		4.1	0.045		64.6		0.000		14.0		0.000		5.0		0.009	2.3		0.060
*G*'_0_ − *G”*_0_ (Pa s*^n^*)	2830.3		0.000		871.3		0.000		461.1		0.000		11.9	0.001		285.7		0.000		24.4		0.000		1306.8		0.000	404.5		0.000
**Parameter**	***T* × *S*** **(*df* = 2)**			***t* × *S*** **(*df* = 2)**				**Pressure × *T* × *t*** **(*df* = 8)**				**Pressure × *T* × *S*** **(*df* = 4)**					**Pressure × *t* × *S*** **(*df* = 4)**				***T* × *t* × *S*** **(*df* = 4)**				**Pressure × *T* × *t* × *S*** **(*df* = 8)**				
	***F* value**	***p* value**		***F* value**		***p* value**		***F* value**		***p* value**		***F* value**			***p*** **value**		***F*** **value**		***p* value**		***F* value**		***p* value**		***F* value**			***p* value**	
*G*’(Pa)	21.7	0.000		134.8		0.000		232.5		0.000		136.0			0.000		198.0		0.000		510.3		0.000		169.9			0.000	
*G”* (Pa)	38.9	0.000		303.0		0.000		401.9		0.000		400.5			0.000		479.4		0.000		1182.9		0.000		444.5			0.000	
tan *δ* (-)	22.9	0.000		44.1		0.054		6.4		0.000		10.4			0.000		12.3		0.000		35.8		0.000		5.5			0.000	
*n*’(-)	0.0	0.961		1.9		0.152		1.6		0.138		0.8			0.552		1.2		0.320		0.3		0.874		0.7			0.682	
*n”* (-)	14.8	0.000		23.4		0.000		1.7		0.119		14.8			0.000		12.5		0.000		8.1		0.000		5.7			0.000	
*G*’_0_ − *G”*_0_ (Pa s*^n^*)	15.0	0.000		99.9		0.000		177.4		0.000		100.1			0.000		145.2		0.000		377.1		0.000		124.2			0.000	

*F* values calculated considering main effects and interactions as fixed parameters. Rheological properties (*G*’: elastic modulus; *G*”: viscous modulus; tan *δ*: loss tangent; *n*’ and *n*”: regression coefficients relating *G*’ or *G*” and frequency (*f*); *G*’_0_ – *G*”_0_, *gel strength*).

**Figure 2:**
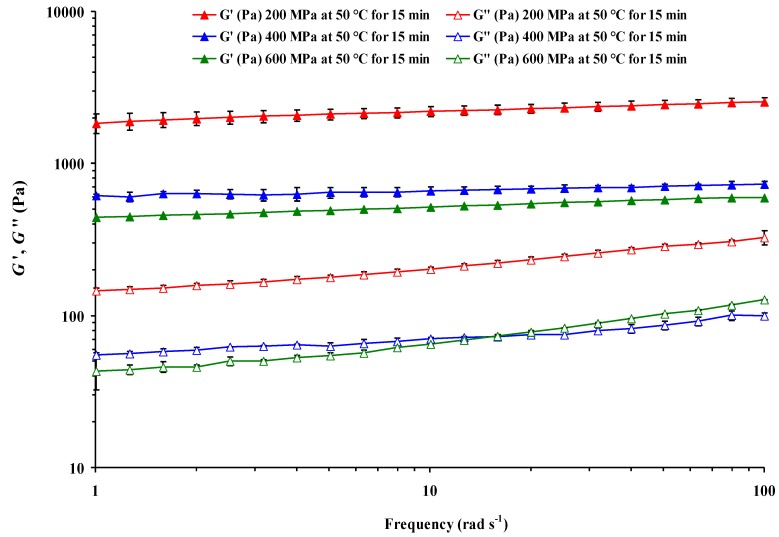
Effect of pressure on mechanical spectra of heat-induced chickpea flour gel pressurized (15 min at 50 °C) at 25 °C. Mean values of six measurements ± error bars.

On the other hand, pressure × *T* interaction was significant for all rheological parameters, whereas the effects of binary pressure × *t*, pressure × *S*, and *T* × *S* interactions were not significant for slope of storage modulus (*n*’). In turn, *T* × *t* interaction was not significant for the slopes of the two moduli (*n*’ and *n*”), whereas *t* × *S* interaction was not significant for *n*’ and loss tangent (tan *δ*). Triple pressure × *T* × *t* interaction only was not significant for the slopes of both *G*’ and *G*”. Consequently, the effect of pressure was dependent on both *T* and treatment *t*, corroborating previous findings [6,7,8]. According to Bauer and Knorr [6], at constant *T* and treatment *t*, the degree of gelatinization increased with increasing pressure. Moreover, the higher the *T*, the lower the pressure of complete gelatinization was in the *T* range investigated (29, 39, 48, and 57 °C), and at constant *T* and pressure the degree of gelatinization of potato, wheat, and tapioca starch suspensions increased with increasing treatment *t* between 5 and 60 min. Stolt *et al*. [8] also showed that in barley starch suspensions rheological properties, microstructure, and loss of birefringence, as well as melting of amylopectin crystals as determined by DSC, were all both pressure- and *t*-dependent. In this study, at a constant 50 °C for 15 min, CF gel rigidity decreased with increasing pressure (Figure 2). This result would also indicate that CF slurry at the highest pressure (600 MPa) had a higher degree of gelatinization during pressurization. The viscoelasticity of thermally-induced CF paste increased as a function of slurry concentration and decreased with increasing pressure applied in proportion to the extent of HHP-induced gelatinization of starch [5]. The authors just cited also found that the gelatinization enthalpies of the HHP-treated CF slurries reflected progressive gelatinization as the pressure level increased, *i.e.*, the enthalpy decreased with increasing pressure applied. However, no starch gelatinization peak was detected during thermal scanning of lentil slurries (untreated or treated) irrespective of moisture content or heating rate [12]. The presence of a significant amount of proteins with sharp endothermic peak could hinder the traceability of a relatively small gelatinization lentil starch peak.

Frequency sweep tests of HHP-treated (Figure 2) and unpressurized samples were carried out in the LVE range after *T* equilibration to 25 °C, which enables the material to retain the structure. The LVE domain was previously established by an oscillatory stress sweep experiment for each sample, as shown previously [5]. For all HHP treatments, *G*’ values were greater than the *G*” values, showing a weak-gel behavior with *G*’ > *G*”, suggesting that elastic properties were dominant over viscous properties (Figure 2). Such behavior has been reported earlier for starch gels thermally induced from basmati rice [11] and HHP-induced CF gels [5].

Both paste and gel may be considered as composite materials, with swollen starch granules filling the polymer solution or polymer gel network, and, if the starch concentration is high enough, the paste can convert into a gel during cooling [2,24]. The viscosity of the paste is the result of the swelling and disintegration of the granules, and the gel formation is the result of the reassociation of the starch macromolecules, especially the amylose, solubilized in the gelatinization process.

**Figure 3:**
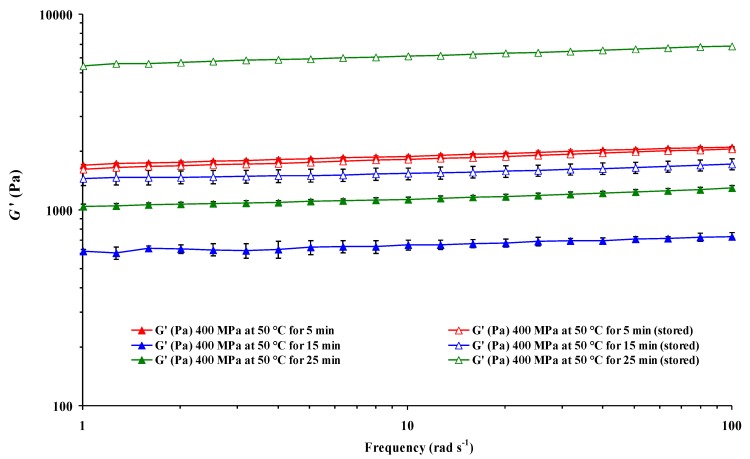
Effect of treatment time and storage on frequency sweeps of elastic modulus (*G*’) of heat-induced chickpea flour gel pressurized (400 MPa at 50 °C) at 25 °C. Mean values of six measurements ± error bars.

In turn, triple pressure × *T* × *S*, pressure × *t* × *S*, *T* × *t* × *S* interactions, and quadruple interaction pressure × *T* × *t* × *S* only were not significant for *n*’ (Table 1). Therefore, the effect of storage was also dependent on pressure and both *T* and treatment *t*. Once more, all the double, triple, and quadruple interactions had a more significant effect on the *G*” values of the heat-induced CF gels after pressurization. As an example, frequency sweeps of *G*’ for CF slurries HHP-treated at 400 MPa and 50 °C for 5, 15, and 25 min are shown in Figure 3, where it is possible to see that the effect of *S* on the *G*’ values at constant pressure and *T* was dependent on treatment *t*.

At 50 °C for 15 and 25 min, *G*’ increased after storage, but decreased after pre-treatment for 5 min. It is possible, therefore, that there was physical aging of starch in HHP-treated slurry samples during storage. An increased rapid retrogradation of leached amylose (as indicated by a higher increase in *G*’ values on cooling) due to disintegration might explain the significant increase in *G*’ upon cooling for CF slurries pre-treated at 400 MPa and 50 °C for 15 and 25 min.

Table 2 and Table 3 show mean values of rheological and power law parameters, respectively, for the CF gels HHP-treated at the various combinations of the four factors studied. Values for unpressurized sample are given as a reference, but they were not included for statistical analyses. As can be seen, for the unpressurized sample only the effect of storage was considered for statistical analysis. The *G*’ and *G*” values for the untreated case without *S* or refrigeration were 3499.0 ± 137.2 and 334.3 ± 5.1 Pa, respectively (Table 2), whereas for refrigerated samples the magnitudes of *G*’ and *G*” were significantly lower, showing that for only *T*-induced gelatinization of CF slurry without previous pressurization both elasticity and viscosity decreased with storage. According to Oey *et al*. [25], the increase in elasticity of the samples treated at the lowest pressure could be attributed to an increase in the linearity of the cell walls and volumes of particles owing to the permeability of the cell walls. In normal rice starch the degree of swelling did not change until the treatment pressure was greater than 300 MPa, and then increased rapidly as the treatment pressure increased up to 500 MPa [26]. The maximum degree of swelling was approximately 50%. Waxy rice starch showed a minor increase in the degree of swelling at treatment pressures below 300 MPa. The degree of swelling then increased very sharply between 300 and 400 MPa and reached 100% at 400 MPa.

**Table 2:** Mean values of rheological properties (*G*’, *G*”, and tan *δ* at 1 Hz) of chickpea flour gel heat-induced at 75 °C for 15 min derived from mechanical spectra after cooling to 25 °C in samples without and with storage for the different pressures (200, 400, and 600 MPa) and treatment temperatures (10 and 25 °C) applied for 5, 15, and 25 min, and for untreated samples.

HHP (MPa)	Storage (*S*)	10 °C								
		5 min			15 min			25 min		
		*G*’ (Pa)	*G*” (Pa)	tan *δ* (-)	*G*’ (Pa)	*G*” (Pa)	tan *δ* (-)	*G*’ (Pa)	*G*” (Pa)	tan *δ* (-)
200	Not stored	1853.3**^C^_b_^2^**	177.8**^C^_a_^2^**	0.10**^A^_a_^1^**	3217.3^B^_a_^1^*	281.2^B^_a_^1^*	0.09**^A,B^_a_^1^**	3770.7^A^_a_^1^*	315.6^A^_a_^1^*	0.08**^B^_a_^1^**
		(68.5)	(3.4)	(0.00)	(141.6)	(4.6)	(0.00)	(137.6)	(3.2)	(0.00)
	Stored	2122.3^B^_b_^1^*	200.9^B^_a_^1^*	0.09**^A^_a_^2^**	2654.7**^A^_b_^1^**	239.5**^A^_b_^1^**	0.09**^A^_a_^1^**	1700.7**^C^_b_^2^**	152.1**^C^_b_^3^**	0.09**^A^_a_^1^**
		(70.1)	(2.4)	(0.00)	(59.0)	(0.4)	(0.00)	(54.2)	(1.8)	(0.00)
400	Not stored	2551.3**^B^_a_^1^**	167.8**^B^_b_^1^**	0.07**^A^_b_^2^**	3184.7**^A^_a_^1^**	195.0**^A^_b_^1^**	0.06**^B^_c_^2^**	1611.7**^C^_b_^1^**	99.1**^C^_c_^2^**	0.06**^B^_b_^2^**
		(124.9)	(2.9)	(0.00)	(149.6)	(3.4)	(0.00)	(58.2)	(5.2)	(0.00)
	Stored	3176.0^C^_a_^1^*	203.5^C^_a_^1^*	0.06**^A^_c_^2^**	5472.0^A^_a_^1^*	355.7^A^_a_^1^*	0.07**^A^_c_^2^**	3588.7^B^_a_^2^*	239.3^B^_a_^2^*	0.07**^A^_c_^1^**
		(89.3)	(0.3)	(0.00)	(118.4)	(6.0)	(0.00)	(78.1)	(1.7)	(0.00)
600	Not stored	720.6**^C^_c_^2^**	64.1**^C^_c_^2^**	0.09^A^_b_^2^*	1491.0**^A^_b_^1^**	124.1**^A^_c_^1^**	0.08**^B^_b_^2^**	1335.7**^B^_c_^1^**	110.2**^B^_b_^2^**	0.08**^B^_a_^2^**
		(20.1)	(3.3)	(0.00)	(44.7)	(5.2)	(0.00)	(29.0)	(4.9)	(0.00)
	Stored	1526.3^A^_c_^1^*	120.7^A^_b_^1^*	0.08**^A^_b_^3^**	1452.3**^B^_c_^1^**	111.5**^B^_c_^1^**	0.08**^B^_b_^2^**	1403.7**^B^_c_^1^**	113.1**^A,B^_c_^2^**	0.08**^B^_b_^2^**
		(33.0)	(2.9)	(0.00)	(21.7)	(4.5)	(0.00)	(28.7)	(3.2)	(0.00)
**HHP** **(MPa)**	**Storage** **(*S*)**	**25 °C**								
		**5 min**			**15 min**			**25 min**		
		***G*’ (Pa)**	***G*” (Pa)**	**tan *δ* (-)**	***G*’ (Pa)**	***G*” (Pa)**	**tan *δ* (-)**	***G*’ (Pa)**	***G*” (Pa)**	**tan *δ* (-)**
200	Not stored	1296.3**^C^_a_^3^**	127.2**^C^_a_^3^**	0.10^A^_a_^1^*	2052.3**^B^_a_^2^**	179.0**^B^_a_^2^**	0.09**^B^_b_^1^**	2727.3**^A^_a_^1,2^**	236.3**^A^_a_^2^**	0.09**^B^_a_^1^**
		(54.4)	(2.4)	(0.00)	(81.1)	(1.5)	(0.00)	(89.4)	(1.7)	(0.00)
	Stored	1704.0^C^_b_^2^*	154.1^C^_a_^2^*	0.09**^A^_b_^2^**	2233.7**^B^_a_^2^**	179.5**^B^_a_^3^**	0.08**^B^_a_^2^**	2706.7**^A^_a_^1^**	229.7**^A^_a_^2^**	0.08**^B^_a_^1^**
		(66.1)	(1.8)	(0.00)	(66.7)	(1.1)	(0.00)	(107.4)	(2.5)	(0.00)
400	Not stored	673.9**^B^_b_^3^**	57.0**^B^_b_^3^**	0.08^B^_b_^1^*	556.4**^A^_c_^2^**	56.4**^B^_c_^3^**	0.10^A^_a,b_^1^*	1723.7**^C^_b_^1^**	114.9**^A^_b_^1^**	0.07**^C^_b_^1^**
		(2.7)	(1.0)	(0.00)	(36.2)	(3.3)	(0.00)	(56.8)	(1.0)	(0.00)
	Stored	2374.7^B^_a_^2^*	151.6^B^_a_^2^*	0.06**^A^_c_^2^**	1338.7^C^_b_^2^*	91.9^C^_c_^3^*	0.07**^A^_b_^1^**	2640.7^A^_a_^3^*	168.9^A^_b_^3^*	0.06**^A^_b_^1^**
		(71.7)	(2.6)	(0.00)	(21.1)	(0.1)	(0.00)	(81.7)	(1.3)	(0.00)
600	Not stored	549.0**^C^_c_^3^**	53.4**^B^_b_^2^**	0.10**^A^_a_^2^**	854.8**^B^_b_^2^**	81.4**^B^_b_^2^**	0.10**^A^_a_^2^**	1218.0**^A^_c_^2^**	104.6**^A^_c_^2^**	0.09**^A^_a_^2^**
		(10.3)	(3.5)	(0.00)	(24.2)	(5.0)	(0.00)	(43.0)	(4.9)	(0.00)
	Stored	842.5^C^_c_^2^*	82.5^B^_b_^2^*	0.10**^A^_a_^2^**	1229.3^A^_c_^2^*	99.4^A^_b_^2^*	0.08**^C^_a_^2^**	1121.7**^B^_b_^3^**	99.1**^A^_c_^3^**	0.09**^B^_a_^2^**
		(8.3)	(1.8)	(0.00)	(21.7)	(2.0)	(0.00)	(31.8)	(2.7)	(0.00)
**HHP** **(MPa)**	**Storage** **(*S*)**	**50 °C**								
		**5 min**			**15 min**			**25 min**		
		***G*’ (Pa)**	***G*” (Pa)**	**tan *δ* (-)**	***G*’ (Pa)**	***G*” (Pa)**	**tan *δ* (-)**	***G*’ (Pa)**	***G*” (Pa)**	**tan *δ* (-)**
200	Not stored	4118.7^A^_a_^1^*	357.9^A^_a_^1^*	0.09**^A^_b_^2^**	2133.7**^C^_a_^2^**	185.6**^C^_a_^2^**	0.09**^A^_b_^1^**	2601.0**^B^_a_^2^**	218.4**^B^_a_^3^**	0.08**^A^_b_^1^**
		(207.0)	(7.7)	(0.00)	(137.6)	(6.7)	(0.00)	(92.8)	(3.6)	(0.00)
	Stored	1242.3**^C^_b_^3^**	122.9**^C^_b_^3^**	0.10^A^_b_^1*^	2204.0**^B^_a_^2^**	187.4**^B^_a_^2^**	0.08**^B^_b_^2^**	2774.0**^A^_b_^1^**	237.2^A^_b_^1^*	0.09**^B^_b_^1^**
		(17.3)	(0.7)	(0.00)	(85.5)	(1.5)	(0.00)	(87.5)	(3.2)	(0.00)
400	Not stored	1845.0^A^_b_^2^*	134.5**^A^_b_^2^**	0.07**^B^_c_^2^**	649.6**^C^_b_^2^**	65.7**^C^_b_^2^**	0.10^A^_b_^1^*	1114.0**^B^_c_^2^**	77.0**^B^_c_^3^**	0.07**^B^_c_^1^**
		(25.5)	(2.2)	(0.00)	(37.8)	(3.2)	(0.01)	(22.7)	(0.3)	(0.00)
	Stored	1771.7**^B^_a_^3^**	150.0**^B^_a_^2^**	0.08^A^_c_^1^*	1510.3^C^_b_^2^*	101.8^C^_b_^2^*	0.07**^B^_c_^1^**	5971.0^A^_a_^1^*	429.8^A^_a_^1^*	0.07**^B^_c_^1^**
		(23.3)	(2.0)	(0.00)	(88.1)	(0.4)	(0.00)	(77.0)	(9.6)	(0.00)
600	Not stored	853.0^B^_c_^1^*	99.6^B^_c_^1^*	0.12**^A^_a_^1^**	470.7**^C^_b_^3^**	59.7**^C^_b_^3^**	0.13**^A^_a_^1^**	1287.7**^A^_b_^1,2^**	152.7**^A^_b_^1^**	0.12**^A^_a_^1^**
		(35.0)	(9.2)	(0.01)	(13.4)	(3.2)	(0.01)	(17.0)	(3.5)	(0.00)
	Stored	295.3**^B^_c_^3^**	44.4**^B^_c_^3^**	0.15^A^_a_^1^*	484.6**^C^_c_^3^**	54.7**^C^_c_^3^**	0.11**^A^_a_^1^**	1256.3**^A^_c_^2^**	134.5**^A^_c_^1^**	0.11**^A^_a_^1^**
		(42.2)	(3.2)	(0.01)	(14.1)	(1.5)	(0.00)	(41.2)	(2.7)	(0.00)
0.1	Not stored	3499.0* (137.2)	334.3* (5.1)	0.10 (0.00)						
	Stored	1874.3 (58.9)	187.4 (1.7)	0.10 (0.00)						

Values between parentheses are standard deviations. ^abc^ For the same column and for the same temperature, time, and parameter, means without the same letter are significantly different (*p* < 0.05) (effect of treatment pressure). ^123^ For the same column and for the same pressure, time, and parameter, means without the same letter are significantly different (*p* < 0.05) (effect of treatment temperature). ^ABC^ For the same row and for the same pressure, temperature, and parameter, means without the same letter are significantly different (*p* < 0.05) (effect of treatment time). * For the same column and for the same pressure, temperature, time, and parameter, the asterisk indicates a significant difference (*p* < 0.05) (effect of storage). Rheological properties (*G*’: elastic modulus; *G*”: viscous modulus; tan *δ*: loss tangent).

**Table 3:** Mean values of power law parameters (*n*’, *n*”, and *G*’_0_ – *G*”_0_ at 1 Hz) from Equations (1) and (2) of chickpea flour gel heat-induced at 75 °C for 15 min derived from mechanical spectra after cooling to 25 °C in samples without and with storage for the different pressures (200, 400, and 600 MPa) and treatment temperatures (10 and 25 °C) applied for 5, 15, and 25 min, and for untreated samples.

HHP (MPa)	Storage (*S*)	10 °C								
		5 min			15 min			25 min		
		*n*’	*n*”	*G*’_0_ − *G*”_0_ (Pa s*^n^*)	*n*’	*n*”	*G*’_0_ − *G*”_0_ (Pa s*^n^*)	*n*’	*n*”	*G*’_0_ − *G*”_0_ (Pa s*^n^*)
200	Not stored	0.07**^A^_a_^1^**	0.18**^A^_b_^2^**	1667.4**^C^_b_^2^**	0.06**^A^_a_^1^**	0.18**^A^_b_^1^**	2926.1^B^_a_^1*^	0.06**^A^_a_^1^**	0.19**^A^_b_^1^**	3443.0^A^_a_^1^*
		(0.01)	(0.00)	(70.2)	(0.01)	(0.01)	(146.6)	(0.00)	(0.00)	(145.3)
	Stored	0.07**^A^_a_^1^**	0.18**^A^_b_^2^**	1919.3^B^_b_^1^*	0.06**^A^_a_^1^**	0.17**^B^_a_^2^**	2408.3**^A^_b_^1^**	0.06**^A^_a_^1^**	0.18**^A^_a,b_^2^**	1545.7**^C^_b_^1^**
		(0.01)	(0.00)	(77.1)	(0.00)	(0.01)	(64.5)	(0.01)	(0.00)	(57.9)
400	Not stored	0.05**^A^_b_^1^**	0.21**^B^_a_^1^**	2375.6**^B^_a_^1^**	0.05**^A^_b_^1^**	0.21^B^_a_^1^*	2980.9**^A^_a_^1^**	0.05**^A^_a_^1^**	0.25^A^_a_^1^*	1511.3**^C^_b_^1^**
		(0.01)	(0.01)	(130.9)	(0.01)	(0.01)	(163.2)	(0.01)	(0.01)	(64.1)
	Stored	0.05**^A^_b_^1^**	0.20**^A^_a_^1^**	2963.7^C^_a_^1^*	0.07**^A^_b_^1^**	0.18**^A^_a_^1^**	5105.8^A^_a_^1^*	0.05**^A^_b_^1^**	0.19**^A^_a_^1^**	3341.7^B^_a_^2^*
		(0.00)	(0.01)	(97.8)	(0.01)	(0.01)	(140.8)	(0.00)	(0.01)	(89.5)
600	Not stored	0.05**^A^_b_^3^**	0.19**^A^_b_^2^**	656.0**^C^_c_^2^**	0.04**^A^_b_^2^**	0.17**^A^_b_^1^**	1366.5**^A^_b_^1^**	0.05**^A^_a_^2^**	0.18**^A^_b_^1^**	1221.5**^B^_c_^1^**
		(0.00)	(0.01)	(18.1)	(0.00)	(0.01)	(43.3)	(0.00)	(0.00)	(28.1)
	Stored	0.04**^A^_b_^2^**	0.17**^A^_b_^2^**	1400.8^A^_c_^1^*	0.04**^A^_b_^2^**	0.18**^A^_a_^2^**	1337.3**^B^_c_^1^**	0.04**^A^_b_^2^**	0.17**^A^_b_^2^**	1287.0**^B^_c_^1^**
		(0.00)	(0.01)	(25.1)	(0.00)	(0.00)	(21.5)	(0.00)	(0.01)	(25.6)
**HHP** **(MPa)**	**Storage** **(*S*)**	**25 °C**								
		**5 min**			**15 min**			**25 min**		
		***n*’**	***n*”**	***G*’_0_ −** ***G*”_0_ (Pa s*^n^*)**	***n*’**	***n*”**	***G*’_0_ −** ***G*”_0_ (Pa s*^n^*)**	***n*’**	***n*”**	***G*’_0_ −** ***G*”_0_ (Pa s*^n^*)**
200	Not stored	0.07**^A^_a_^1^**	0.20**^A^_b_^1^**	1163.3**^C^_a_^3^**	0.06**^A^_a_^1^**	0.19**^A^_a_^1^**	1872.1**^B^_a_^2^**	0.06**^A^_a_^1^**	0.19**^A^_b_^1^**	2487.0**^A^_a_^2^**
		(0.00)	(0.00)	(56.5)	(0.01)	(0.01)	(83.6)	(0.01)	(0.00)	(96.9)
	Stored	0.06**^A^_a_^1^**	0.19**^A^_a_^1^**	1548.4^C^_b_^2^*	0.06**^A^_a_^1^**	0.20**^A^_a_^1^**	2052.0**^B^_a_^2^**	0.06**^A^_a_^1^**	0.19**^A^_b_^1^**	2469.3**^A^_a_^2^**
		(0.01)	(0.00)	(69.1)	(0.01)	(0.00)	(73.5)	(0.01)	(0.00)	(113.8)
400	Not stored	0.06**^A^_b_^1^**	0.21**^A^_b_^1^**	617.2**^B^_b_^3^**	0.04**^A^_b_^1^**	0.19**^B^_a_^2^**	501.0**^A^_c_^2^**	0.05**^A^_a,b_^1^**	0.22^A^_a_^2^*	1604.4**^C^_b_^1^**
		(0.00)	(0.00)	(4.1)	(0.01)	(0.00)	(36.6)	(0.01)	(0.00)	(63.0)
	Stored	0.05**^A^_b_^1^**	0.19**^B^_a_^1^**	2213.6^B^_a_^2^*	0.07**^A^_a_^1^**	0.20**^A,B^_a_^1^**	1247.1^C^_b_^2^*	0.05**^A^_b_^1^**	0.21**^A^_a_^1^**	2461.2^A^_a_^3^*
		(0.01)	(0.01)	(79.0)	(0.00)	(0.00)	(25.4)	(0.01)	(0.00)	(87.6)
600	Not stored	0.06**^A^_b_^2^**	0.23^A^_a_^1^*	498.3**^A^_c_^3^**	0.05**^B^_b_^2^**	0.19**^A^_a_^1^**	773.1**^B^_b_^2^**	0.04**^B^_b_^2^**	0.19^A^_b_^1^*	1114.1^C^_c_^2^*
		(0.00)	(0.01)	(5.71)	(0.00)	(0.02)	(18.5)	(0.00)	(0.01)	(37.0)
	Stored	0.05**^A^_a,b_^1,2^**	0.15**^B^_b_^2^**	753.9^C^_c_^2^*	0.05**^A^_a_^2^**	0.19**^A^_a_^2^**	1127.9^A^_c_^2^*	0.05**^A^_b_^2^**	0.16**^B^_c_^2^**	1020.2**^B^_b_^3^**
		(0.00)	(0.01)	(7.6)	(0.00)	(0.01)	(13.0)	(0.00)	(0.01)	(30.1)
**HHP** **(MPa)**	**Storage** **(*S*)**	**50 °C**								
		**5 min**			**15 min**			**25 min**		
		***n*’**	***n*”**	***G*’_0_ −** ***G*”_0_ (Pa s*^n^*)**	***n*’**	***n*”**	***G*’_0_ −** ***G*”_0_ (Pa s*^n^*)**	***n*’**	***n*”**	***G*’_0_ −** ***G*”_0_ (Pa s*^n^*)**
200	Not stored	0.07**^A^_a_^1^**	0.16**^C^_b_^3^**	3732.5^A^_a_^1*^	0.07**^A^_a_^1^**	0.18**^A^_a_^1^**	1931.8**^C^_a_^2^**	0.06**^A^_b_^1^**	0.17**^B^_b_^2^**	2371.7**^B^_a_^2^**
		(0.01)	(0.00)	(226.2)	(0.01)	(0.00)	(148.4)	(0.01)	(0.00)	(98.2)
	Stored	0.06**^A^_a_^1^**	0.16**^B^_b_^3^**	1116.1**^C^_b_^3^**	0.06**^A^_a_^1^**	0.17**^A^_b_^2^**	2014.8**^B^_a_^2^**	0.06**^A^_b_^1^**	0.16**^B^_b_^3^**	2525.4**^A^_b_^2^**
		(0.00)	(0.00)	(21.5)	(0.00)	(0.00)	(86.0)	(0.01)	(0.00)	(93.8)
400	Not stored	0.05**^A^_b_^1^**	0.19^B^_a,b_^2^*	1701.6^A^_b_^2^*	0.04**^A^_b_^1^**	0.12**^C^_b_^3^**	551.2**^C^_b_^2^**	0.05**^A^_c_^1^**	0.21^A^_a_^2^*	1037.8**^B^_b_^2^**
		(0.00)	(0.01)	(20.9)	(0.00)	(0.01)	(29.8)	(0.01)	(0.01)	(21.1)
	Stored	0.05**^A^_b_^1^**	0.16**^A^_b_^2^**	1620.5**^B^_a_^3^**	0.07**^B^_b_^1^**	0.18^A^_b_^1^*	1416.6^C^_b_^2^*	0.05**^A^_c_^1^**	0.16**^A^_b_^2^**	5524.3^A^_a_^1^*
		(0.00)	(0.01)	(23.4)	(0.01)	(0.02)	(91.9)	(0.01)	(0.01)	(89.6)
600	Not stored	0.08**^A^_a_^1^**	0.21**^A^_a_^1,2^**	755.2^B^_c_^1^*	0.07**^A^_a_^1^**	0.19**^A^_a_^1^**	407.4**^C^_b_^3^**	0.08**^A^_a_^1^**	0.20**^A^_a_^1^**	1124.6**^A^_b_^2^**
		(0.01)	(0.02)	(37.3)	(0.01)	(0.01)	(7.2)	(0.01)	(0.01)	(26.2)
	Stored	0.06**^A^_a_^1^**	0.22**^B^_a_^1^**	248.4**^B^_c_^3^**	0.07**^A^_a_^1^**	0.23^A^_a_^1^*	430.0**^C^_c_^3^**	0.07**^A^_a_^1^**	0.21**^B^_a_^1^**	1110.4**^A^_c_^2^**
		(0.01)	(0.01)	(40.0)	(0.01)	(0.01)	(13.4)	(0.00)	(0.01)	(41.6)
0.1	Not stored	0.07 (0.01)	0.16 (0.00)	3149.8* (147.5)						
	Stored	0.07 (0.00)	0.18 (0.01)	1684.3 (64.0)						

Values between parentheses are standard deviations. ^abc^ For the same column and for the same temperature, time, and parameter, means without the same letter are significantly different (*p* < 0.05) (effect of treatment pressure). ^123^ For the same column and for the same pressure, time, and parameter, means without the same letter are significantly different (*p* < 0.05) (effect of treatment temperature). ^ABC^ For the same row and for the same pressure, temperature, and parameter, means without the same letter are significantly different (*p* < 0.05) (effect of treatment time). * For the same column and for the same pressure, temperature, time, and parameter, the asterisk reveals significant difference (*p* < 0.05) (effect of storage). Rheological properties (*n*’ and *n*”: regression coefficients relating *G*’ or *G*” and frequency (*f*); *G*’_0_ − *G*”_0_, *gel strength*).

For refrigerated or stored samples the range of variation of both *G*’ and *G*” was wider, ranging from 295.3 ± 42.2 to 5971.0 ± 77.0 Pa and from 44.4 ± 3.2 to 429.8 ± 9.6 Pa, respectively. The lowest *G*’ and *G*” values were obtained for stored CF slurries treated with 600 MPa at 50 °C for 5 min, but the highest ones corresponded to the refrigerated CF slurries treated with 400 MPa at 50 °C for 25 min. A previous study also showed that complete gelatinization (100%) was associated with CF slurries at the lowest concentration (1:5) after HHP with 600 MPa at 25 °C for 15 min [5]. Clearly, other additional structural features seem to be responsible for the altered *G*’ and *G*” values obtained for the samples pre-treated with 400 and 600 MPa at 50 °C for 25 min. Results would appear to indicate that breakdown of granules and some splitting of macromolecules could be assumed at 400 and 600 MPa and higher *T* and *t*s. In these cases, retrogradation probably occurred outside the granules, explaining the higher parameter values obtained for the CF slurries HHP-treated at 400 MPa and 50 °C for 25 min. Other publications showed the presence of a residual crystalline order after pressure treatment, referred to as “rapid retrogradation” occurring even during or immediately after pressurization [4,10], and the greater the degree of gelatinization induced by the pressure treatment, the greater the extent of “rapid retrogradation”. On the other hand, denaturation of protein component could also affect the rheological properties of these samples.

On the other hand, at constant *T* and treatment *t*, for non-stored HHP-treated CF slurries both *G*’ and *G*” values tended to decrease with increasing pressure (Table 2). At 10 °C for 5 min, *G*’ increased between 200 and 400 MPa, but decreased after pre-treatment with 600 MPa. In contrast, for CF slurries pre-treated at 25 °C for 15 min and at 50 °C for 25 min, both *G*’ and *G*” values decreased between 200 and 400 MPa, but increased significantly after treatment with 600 MPa, the increase being more significant at the higher *T*. This result might reflect the presence of an appreciable number of swollen starch granules in the CF slurries after these HHP treatments at 600 MPa, which prevent further swelling.

The effects of increasing *T* are essentially energy and volume effects due to thermal expansivity [26]. For unrefrigerated samples, at 200 and 400 MPa for 5 and 15 min, there was a significant decrease in *G*’ and *G*” (Table 2) when *T* was increased from 10 to 25 °C, but both moduli increased again when *T* was increased to 50 °C, especially at the shortest *t*. At 200 MPa for 25 min, *G*’ and *G*” decreased with increasing treatment *T*. Conversely, after pressurization at 400 MPa for 25 min, both *G*’ and *G*” values increased when *T* was increased from 10 to 25 °C, but decreased at 50 °C. For the CF gels induced from unrefrigerated samples pressurized at 600 MPa for 5 min, *G*’ and *G*” decreased when *T* was increased from 10 to 25 °C, but increased at 50 °C. At 600 MPa for 15 min, the *G*' and *G*" values decreased significantly and the tan *δ* values increased with *T*. Finally, at 600 MPa for 25 min, both *G*’ and *G*” values decreased when *T* was increased from 10 to 25 °C, but increased at 50 °C.

Therefore, increasing *T* from 10 to 50 °C increased the degree of gelatinization of the slurries, especially of those pressurized at 200 and 400 MPa for 25 min and at 600 MPa for 15 min, and consequently the *T*-induced CF gels subsequently had inferior mechanical strength. However, the *T* effect was more significant at a constant pressure of 600 MPa and *t* of 15 min. Note that at 600 MPa for 5, 15, and 25 min there was a significant increase in tan *δ* when *T* was increased from 10 to 50 °C. This would represent a less structured system at the highest *T*, as can be observed from the significantly greater values of loss tangent [27]. On the other hand, tan *δ* = *G*”/*G*’ values were significantly lower than 0.5 in all the *T*-induced CF gels without and after pressurization for the various combinations, meaning that the samples behave like a viscoelastic gel because *G*’ is larger than *G*”, indicating the presence of a network structure [28]. For normal rice starch, at 400 MPa the degree of gelatinization (*η*_initial_) increased gradually as the temperature was increased from 10 to 60 °C [26], whereas for waxy rice starch, at 350 MPa *η*_initial_ increased gradually as the temperature was increased from 20 to 60 °C.

With regard to the treatment *t* effect, at constant pressure and *T*, unrefrigerated samples HHP-treated at 200 and 600 MPa and 10 °C, at 200, 400, and 600 MPa and 25 °C, and at 600 MPa and 50 °C showed a significant increase in both *G*’ and *G*” values (Table 2), with increasing treatment *t* from 5 up to 25 min, resulting in an increase in CF paste and gel rigidity, confirming that HHP-induced gelatinization is also a *t*-dependent process [6]. This result could indicate an association between amylose and amylopectin induced by pressurization throughout these different HHP treatment combinations, which produced a cross-linking-like network that displayed resistance to shear force and increased viscosity [16]. As mentioned previously, for the slurries pre-treated at 200 MPa and 10 °C for 25 min without previous *S*, the *G*’ value (3770.7 ± 137.6 Pa) was higher than the value achieved by the unpressurized sample, indicative of its higher ability for *T*-induced gelatinization even after pressurization at the lowest pressure and *T* for 25 min. Similarly, CF samples containing a flour-to-water ratio of 1:5 that were pre-treated at 150 MPa (at 25 °C for 15 min) and then heated under isothermal conditions up to 75 or 90 °C had significantly higher *G*’ and *G*” values than the unpressurized case, suggesting that HHP-induced melting of granules started at pressures >150 MPa [5].

Conversely, at constant pressure and *T*, unrefrigerated samples HHP-treated at 400 MPa and 10 °C, as well as at 200 and 400 MPa and 50 °C, showed a significant decrease in both *G*’ and *G*” valueswith increasing treatment *t* from 5 up to 25 min. In addition, a significant decrease in both *G*’ and *G*” values was observed with increasing treatment *t* from 5 up to 15 min for CF slurries pre-treated at 200, 400, and 600 MPa and 50 °C, whereas the contrary was true after pressurization at the same pressures and 10 °C.

Table 2 also shows the effect of pressure, *T*, and treatment *t* on the rheological properties of heat-induced CF gels after keeping the HHP-treated CF slurries for one week under refrigeration or storage at 4 °C. At constant *T* and treatment *t*, for CF slurries pre-treated at 25 °C for 15 and 25 min and at 50 °C for 5 and 15 min, both *G*’ and *G*” values decreased significantly with increasing pressure applied, reflecting progressive gelatinization as the pressure level increased from 400 to 600 MPa for these *T* and treatment *t* combinations. On the other hand, the low *G*’ and *G*” values obtained for the CF gels after pressurization at 600 MPa and 50 °C would appear to indicate that the starch had been completely gelatinized by the HHP treatment at 600 MPa and 50 °C for 5 and 15 min. Samples of sorghum starch pre-treated at 500 and 600 MPa at 20 °C for 10 min showed no increase in viscosity, and therefore a complete gelatinization induced by HHP treatment was assumed [9]. Likewise, also after pressurization with 500 MPa, gelatinization was nearly complete and only a very small increase in complex modulus occurred upon pasting [29]. Moreover, the onset complex modulus increased sharply between 300 and 500 MPa and exhibited a sigmoidal-shaped curve. Nevertheless, for the other *T* and treatment *t* combinations the values of both *G*’ and *G*” increased when the pressure increased from 200 to 400 MPa, but also decreased when the pressure increased to 600 MPa.

With regard to the treatment *T* effect on stored CF slurries, at constant pressure and *t*, samples HHP-treated at 200, 400, and 600 MPa for 5 and 15 min showed a significant decrease in their viscoelastic properties (Table 2) with increasing treatment *T* from 10 up to 50 °C, resulting in a reduction in CF paste and gel rigidity, confirming that HHP-induced gelatinization is also a *T*-dependent process [6,7]. In accordance with Stolt *et al*. [8], the same degree of gelatinization can be brought about by different pressure–*T* combinations, *i.e.*, lower pressure can, to a certain extent, be compensated for by higher treatment *T*. Conversely, different results were obtained for the samples HHP-treated at the longest *t*. For the samples HHP-treated at 200 and 400 MPa for 25 min, both *G*’ and *G*” values were significantly higher at 50 than at 10 °C, and the same was true for the viscosity of the CF slurries pressurized at 600 MPa for 25 min. According to Oh *et al*. [7], this result might indicate that a breakdown of granules caused an increase in elasticity and viscosity of refrigerated samples HHP-treated at 200, 400, and 600 MPa and 50 °C for 25 min, with significant leaching of amylose, and therefore a higher extent of recrystallization occurring outside the starch granules. As the quantity of amylose surrounding starch granules was higher in this case, CF gels obtained using this high *T* and treatment *t* had a stronger matrix. Similarly, pressure treatment at 650 MPa for 6 min resulted in a complete breakdown of granules in waxy corn starch, whereas high-amylose corn starch retained a granular structure [30].

With regard to the effect of treatment *t* on the viscoelastic properties of the refrigerated CF slurries, at constant pressure and *T*, samples HHP-treated at 200, 400, and 600 MPa and 50 °C showed a significant increase in both *G*’ and *G*” values with increasing treatment *t* from 5 up to 25 min. Therefore, it is possible that these more severe HHP treatments at the highest *T* and longest *t* would result in a greater degree of breakdown of the granules in the CF slurries.

During gelation, double helices are formed between the leached amylose molecules, and a network develops. This network is reinforced by the embedded deformed gelatinized granules. Upon storage of starch gels, recrystallization occurs: owing to the reassociation and crystallization of starch chains, a new semi-crystalline structure is formed. This reassociation is referred to as retrogradation [31]. Starch retrogradation was found to be the major factor affecting stability and varied greatly among different starches and starch fractions [32,33]. The newly formed crystals exhibit a B-pattern, independent of the crystalline pattern of the native starch [34]. Recrystallization is a slow, two-phase process. The short-term (~1–2 days) recrystallization is dominated by crystallization within the amylose matrix. The double helices form very stable crystals, as they have a melting temperature of about 150 °C [35]. Amylopectin retrogradation is much slower (∼10 days) and the crystals have a lower thermal stability, melting between 50 and 60 °C [34].

The retrogradation of pressure-induced barley starch gels was studied using DSC [8], because it had been reported that pressure-induced starch gels in general are less sensitive to retrogradation than heat-induced gels [13]. In order to investigate the starch retrogradation of HHP-treated CF slurries, at each combination of pressure, *T*, and treatment *t* the subsequent *T*-treatment for inducing CF gels was carried out twice, immediately after HHP treatment and after 1 week of storage at 4 °C. Both *G*’ and *G*” values were significantly higher for the unpressurized CF gel without storage as compared with the unpressurized sample after refrigeration (Table 2). Initially, this decrease may only be associated with sedimentation, flocculation, or coalescence phenomena occurring in the unpressurized CF slurry during storage. In this study, retrogradation from starch gelatinization by heat is discounted because, although the HHP-treated CF slurries were cooled to 25 °C before measurements, there was no time for gel aging.

In addition, both storage and loss moduli also decreased with storage for the CF slurries pre-treated with 200 MPa at 10 °C for 15 and 25 min and at 50 °C for 5 min, as well as for the CF slurries pre-treated with 400 and 600 MPa at 50 °C for 5 min. Conversely, for CF slurry pre-treated with 200, 400, and 600 MPa at 10 and 25 °C for 5 min, with 400 MPa at 10, 25, and 50 °C for 15 and 25 min, and with 600 MPa at 25 °C for 15 min, both *G*’ and *G*” values were significantly higher for the heat-induced CF gels after storage. As the retrogradation process takes place, the starch paste becomes increasingly opaque and could form a cuttable gel. With time, this gel becomes rubbery and has a tendency to release water [31]. In native corn starch an increase in the critical shear stress and a decrease in the critical shear strain were found, which reflected the presence of a stiffer system but one that was more sensitive to the stress applied. According to Kapri and Bhattacharya [2], during retrogradation there is a progressive change from elasto-viscoplastic behavior (presence of a yield stress) to an elastic gel with viscous damping. The events occurring during gelatinization and recrystallization of a starch suspension, especially the granule swelling and leaching of amylose/amylopectin, will strongly change the rheological properties of a starch suspension. It has also been stated that pressure-treated samples exhibited a relatively lower extent of recrystallization compared with thermally treated dispersion during storage, suggesting that less retrogradation occurred in the pressurized starch than in the heated starch [8,9,13,29]. Therefore the increase in the moduli of the HHP-treated CF slurries is probably a consequence of the retrogradation phenomena dominated by crystallization within the amylose matrix occurring during the storage period. However, further research is necessary to explain why both *G*’ and *G*” values were lower in the stored HHP-treated CF slurries for some combinations of the factors.

On the other hand, there were non-significant differences between the viscoelastic properties of refrigerated and unrefrigerated CF slurries HHP-treated at 600 MPa and 50 °C for both 15 and 25 min (Table 2). Results would appear to indicate that breakdown of granules and some splitting of macromolecules could be assumed at 600 MPa and higher *T* and *t*s. In these cases, retrogradation probably occurred outside the granules, which explains the lack of effect of storage on the parameter values obtained for the CF slurries HHP-treated at 600 MPa and 50 °C for both 15 and 25 min. However, to obtain a more detailed understanding of this difference in behavior, further research is necessary between 400 and 600 MPa, as well as between 25 and 50 °C. The information gathered from rheological measurements needs to be related to other analyses to characterize the HHP-induced gelatinization of CF slurry.

Table 3 shows the effects of pressure, *T*, treatment *t*, and *S* on the power law parameters of heat-induced CF gels without and with storage. Exponents *n*’ and *n*” may be related to the time-stability of the network, given the frequency-dependence (*ω*) of viscoelastic moduli [36]. The *n*’ values ranged between 0.04 ± 0.00 and 0.08 ± 0.01, reflecting that for all the CF gels *G*’ is practically frequency-independent over this time range (*n*’ < 0.1) [37]. The highest *n*’ values were obtained for CF slurries treated with 600 MPa at 50 °C for 5 and 25 min. This indicates a less time-stable matrix, resulting in a more shear-strain sensitive network for these CF gels. Note that, in fact, storage was not significant for *n*’ values (Table 1). In turn, the *n*” values ranged between 0.12 ± 0.01 and 0.25 ± 0.01, therefore reflecting a higher *ω* dependence associated with *G*”. For unrefrigerated samples, a significant increase in *n*” was observed with increasing pressure level from 200 to 400 MPa for CF gels pre-treated at 10 °C for 5, 15, and 25 min and at 25 °C for 25 min. Moreover, after 600 MPa at 25 °C for 5 min, CF gel had a significantly higher *n*” than after 200 and 400 MPa, showing that the time-stability of the network decreased. Furthermore, at 400 MPa and 10 °C, *n*” increased significantly with *t* (Table 3).

*S* also had a significant effect on the *n*” values of CF gels pre-treated at 400 MPa and 10 °C for 15 and 25 min, at 400 MPa and 25 °C for 25 min, at 400 MPa and 50 °C for 5, 15, and 25 min, at 600 MPa and 25 °C for 5 min and 25 min, and at 600 MPa and 50 °C for 15 min. Except for HHP treatments at 400 and 600 MPa and 50 °C for 15 min, the *n*” values were lower in the refrigerated samples, indicating a greater degree of connectivity, related to starch retrogradation occurring in these HHP-treated slurry samples during *S*.

The *gel strength* (*G*’_0_ – *G*”_0_) parameter depends on the cross-linking density and the molecular chain [19]. This parameter can be used to assess the firmness of gels upon subjection to a fairly rapid deformation such as depressing the gel quickly with one’s thumb and immediately releasing the pressure. It is worth emphasizing that for both unrefrigerated and refrigerated samples, at 10 °C for 5, 15, and 25 min, *gel strength* increased between 200 and 400 MPa, but decreased to a minimum after pre-treatment with 600 MPa (Table 3). Furthermore, for samples without storage, at 25 °C for 5 and 25 min, and at 50 °C for 5, 15, and 25 min, *gel strength* decreased significantly with pressure, mainly owing to HHP-induced gelatinization of starch. On the other hand, when the samples were pressurized at 400 and 600 MPa and 50 °C, there were no significant differences between the *G*’_0_ – *G*”_0_ values of the gels obtained from the unrefrigerated slurries pressurized for 15 and 25 min. Conversely, for refrigerated samples, at 50 °C for 15 min, the *gel strength* decreased significantly to a minimum after pre-treatment with 600 MPa. This result would reflect that after pressurization at 50 °C for 15 min pressure-induced melting of the granules started at 200 MPa, and complete gelatinization was obtained after treatment with 600 MPa. The relationship between degree of gelatinization and treatment pressure followed a sigmoid-shaped curve [9]. Such sigmoid curves were previously observed for the degree of gelatinization *vs.* pressure, based on the loss of birefringence [6] and on the swelling index [13]. A sigmoidally-shaped gelatinization curve means that pressure-induced gelatinization occurs within a HHP range [4], and that the treatment pressure has to be above a critical level for gelatinization to occur effectively [26].

With regard to the treatment *T* effect, at constant pressure and treatment *t*, both unrefrigerated and refrigerated samples HHP-treated at 400 and 600 MPa for 15 and 25 min mostly showed a significant decrease in *gel strength* (Table 3) with increasing treatment *T* from 10 up to 50 °C, resulting in a reduction in CF paste and gel rigidity, confirming that HHP-induced gelatinization is also a *T*-dependent process [6,8]. Upon further increase in *T*, the granules begin to break down and amylopectin solubilization increases, resulting in a decrease in consistency and overall stiffness [38]. However, for samples HHP-treated at 600 MPa for 25 min, there were no significant differences between the *G*’_0_ − *G*”_0_ values of the gels obtained from the CF slurries pressurized at 25 and 50 °C. This result seems to show that after 600 MPa at 25 °C for 25 min complete gelatinization was obtained, suggesting that, at least at the highest pressure applied, the relationship between degree of gelatinization and treatment *T* also follows a sigmoid-shaped curve.

The importance of treatment duration on starch gelatinization has been highlighted in a number of studies [4,6,8]. In this study, at constant pressure and *T* the effect of treatment *t* on *gel strength* was more ambiguous. Samples without storage treated at 200 and 600 MPa and 10 °C, and at 200, 400, and 600 MPa and 25 °C showed a significant increase in *G*’_0_ – *G*”_0_ values with increasing treatment *t*. However, at the highest *T* a weaker-structured matrix was obtained after pressurization at 200, 400, and 600 MPa for 15 min and subsequently the *gel strength* increased with longer duration. Stolt *et al*. [8] observed that the consistency of a starch–water suspension increased with increasing processing time until a plateau was reached where the consistency did not change further. The more severe the treatment conditions (higher temperature and/or pressure), the faster this plateau value was reached. The use of starch gelatinization as a pressure–time–temperature indicator, an indicator of the severity of the treatment, for monitoring high pressure processing conditions has been discussed by Bauer and Knorr [6].

### 3.2. Effect of Pressure, Temperature (T), and Treatment Time (t) on Textural Properties of CF Gel Heat-Induced from Pressurized Powder Form

All the CF samples that were pressurized in powder form turned into solid rock; therefore, they were hammered into smaller pieces to obtain a free-flowing powder. A similar observation was reported by Katopo *et al*. [16]. Normal maize, waxy maize, high-amylose maize VII, tapioca, potato, and rice starches were ultrahigh hydrostatic pressurized at 690 MPa in powder form by the authors just cited.

With regard to textural properties derived from *BS* and TPA tests for the heat-induced CF gels made with the different pressurized powders, the three-way mixed-model ANOVA showed significant (*p* < 0.05) pressure, *T*, and treatment *t* main effects for rupture strength (*RS*) from the *BS* test and especially for hardness (*Hard*) from the TPA test (Table 4). The effect of *T* was not significant for *Adh_BS_* and *Slope* (brittleness) from the *BS* test, and *Adh_TPA_* and *Coh* from the TPA test, whereas *t* main effect was only significant for *RS*, *Slope*, and *Hard*. Except for *RS* and *Hard*, pressure had a more significant effect on textural properties than either treatment *T* or holding *t*. Katopo *et al*. [16] also found that results obtained from samples with 1 h of pressurization were not significantly different from those obtained with 5 min of pressurization at 690 MPa, indicating that holding *t* had a non-significant effect in their study.

On the other hand, pressure × *t* interaction was not significant for *RS* and *Adh_BS_* from the *BS* test, and pressure × *T* × *t* interaction was only not significant for *Adh_BS_*, whereas the effect of binary pressure × *T* interaction was not significant for *BS*, *Adh_BS_*, and *Adh_TPA_*. In turn, double *T* × *t* interaction was not significant for *BS*, *Adh_BS_, Slope*, and *Adh_TPA_*. ANOVA also showed that all three main effects and all the interactions were not a significant (*p* > 0.05) source of variation for the textural property *Adh_BS_* from the *BS* test. For this type of food, measurement of adhesiveness by the *BS* method is not appropriate. Hoseney and Smewing [39] stated that in order to study adhesive properties it is imperative to have a procedure that forces a clean separation at the probe–material interface.

**Figure 4:**
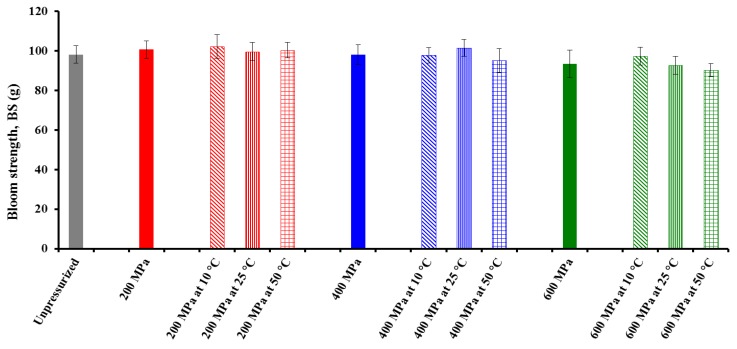
Effect of pressure and treatment temperature on bloom strength (*BS*) of chickpea gel heat-induced from pressurized powder at 25 °C. Mean values of 10 measurements.

**Figure 5:**
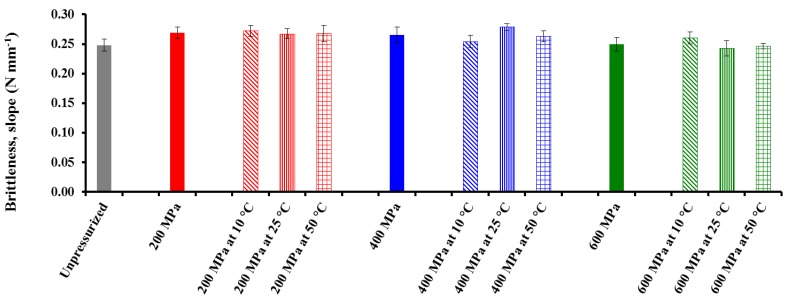
Effect of pressure and treatment temperature on the brittleness (slope) of chickpea gel heat-induced from pressurized powder at 25 °C. Mean values of 10 measurements.

**Figure 6:**
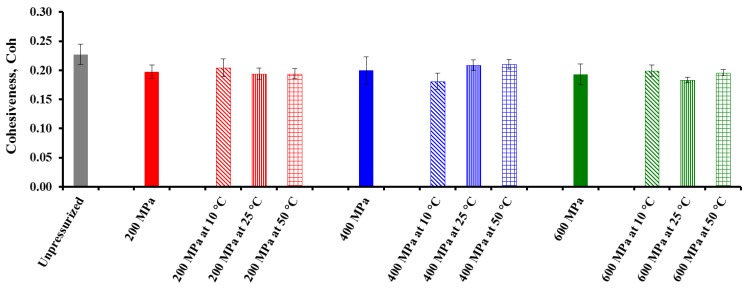
Effect of pressure and treatment temperature on the cohesiveness (*Coh*) of chickpea gel heat-induced from pressurized powder at 25 °C. Mean values of 10 measurements.

As a result, except for *Adh_BS_* from the *BS* test, the effect of pressure on the textural properties of CF gel heat-induced from HHP-treated powder was dependent on both *T* and treatment *t*, although the three main effects and their interactions had a more significant effect on the rheological properties of the HHP-treated CF slurries (Table 1). Graphs of three different textural properties are shown in Figure 4, Figure 5 and Figure 6. Pressure main effect without considering *T* and treatment *t* main effects, as well as binary pressure × *T* interactions discarding the treatment *t* effect, were chosen for representation because their *F* values were mostly higher than those of the triple interactions, and compared with an untreated sample.

The effects of pressure are mainly volume effects caused by compressibility of the system [26]. It can be observed that the *BS* of the CF gels decreased with increasing pressure level, reflecting the fact that treatment of CF in powder form with increasing pressure causes an increase in starch gelatinization, in agreement with previous findings in other either starch- or flour-in-water suspensions [5,6,7,9,29]. More interestingly, samples treated at 600 MPa showed a substantial decrease in their *BS* values as compared with unpressurized powder.

**Table 4:** Three-way analysis of variance of the textural properties of CF gel heat-induced from pressurized powder form after cooling to 25 °C (high hydrostatic pressure treatments: 200, 400, and 600 MPa, 10, 25, and 50 °C, 5, 15, and 25 min, and unpressurized sample). *F* and *p* values.

Parameter	Pressure (*df* = 2)		Temperature (*T*) (*df* = 2)		Time (*t*) (*df* = 2)		Pressure × *T* (*df* = 4)		Pressure × *t* (*df* = 4)		*T* × *t* (*df* = 4)		Pressure × *T* × *t* (*df* = 8)	
	*F* value	*p* value	*F* value	*p* value	*F* value	*p* value	*F* value	*p* value	*F* value	*p* value	*F* value	*p* value	*F* value	*p* value
BS (g)	16.7	0.000	4.8	0.009	2.0	0.136	1.9	0.109	9.5	0.000	1.4	0.251	2.5	0.014
RS (N)	7.2	0.001	4.0	0.020	18.6	0.000	10.5	0.000	1.1	0.354	30.3	0.000	20.0	0.000
Adh_BS_ (N s)	1.1	0.338	1.5	0.232	2.0	0.134	1.4	0.247	1.2	0.322	1.7	0.143	1.7	0.100
Slope (N mm^−1^)	16.7	0.000	1.5	0.216	4.9	0.008	6.4	0.000	4.6	0.001	2.3	0.057	5.5	0.000
Hard (N)	46.2	0.000	66.5	0.000	38.1	0.000	51.2	0.000	60.0	0.000	21.2	0.000	55.0	0.000
Adh_TPA_ (N s)	6.1	0.003	0.1	0.873	1.4	0.253	2.2	0.066	9.6	0.000	0.4	0.817	8.1	0.000
Spr (-)	7.5	0.001	5.1	0.007	1.6	0.209	4.6	0.001	5.5	0.000	2.8	0.025	6.7	0.000
Coh (-)	1.2	0.292	0.8	0.450	0.4	0.702	5.5	0.000	5.2	0.000	4.4	0.002	2.9	0.004

*F* values calculated considering main effects and interactions as fixed parameters. Textural properties (*BS*: weight needed by the probe to deflect the surface of the gel 4 mm without breaking from bloom strength test; *RS*: the rupture strength as the maximum force; *Adh_BS_*: the adhesiveness as the negative area during the probe reversal; *Slope*: brittleness as the slope at maximum force from bloom strength test; *Hard*: hardness from texture profile analysis (TPA); *Adh_TPA_*: adhesiveness from TPA; *Spr*: springiness from TPA; *Coh*: cohesiveness from TPA).

Stolt *et al*. [8] found a complete loss of birefringence in barley starch after HHP treatment at 600 MPa for 15 min. HHP-induced melting of sorghum starch granules started at pressures >300 MPa and complete gelatinization was obtained after treatment with 600 MPa [9]. However, the HHP-gelatinization in the presence of excess water is primarily depended on the applied pressure. The pressure range in which HHP gelatinization of starches can be achieved seems to be between 400 to 900MPa (400 MPa, e.g., for wheat starch and 800–900 MPa for potato starch) [10]. On the other hand, both starch and protein are the major chickpea components. Therefore, in this study, the presence of proteins might have also been responsible for the lower BS values of the CF gels with increasing pressure level. HHP treatment has been shown to influence the functional properties of proteins through the disruption and reformation of hydrogen bonds and hydrophobic interactions leading to denaturation, aggregation, and gelation [11]. The authors just cited inferred that the observed decrease in enthalpy for rice slurry after pressure treatment cannot be attributed solely to starch gelatinization or unfolding of proteins; rather, it results from combination of these components. These observations were supported by DSC data and the sodium dodecyl sulfate polyacrylamide gel electrophoresis (SDS-PAGE) indicated alterations in protein structures following HHP treatment. In turn, Fourier-transform infrared (FTIR) spectroscopy indicated a change in the secondary structure of post-process rice proteins. In any case, CF slurries subjected to 600 MPa at 25 °C for 15 min showed no peak and hence no enthalpy value, suggesting complete HHP-induced gelatinization of starch [5].

Moreover, at constant pressure of 600 MPa the higher the *T*, the lower the *BS* in the *T* range investigated (10, 25, and 50 °C), showing that the degree of gelatinization also increased with increasing treatment *T*. For this reason, the bloom strength (*BS*) of the CF gels obtained fell with increasing pressure. The gelling ability of chickpea flour in powder form and the viscous nature of cooked paste are important for the manufacture of gelled food products [2]. At constant 200 MPa, samples HHP-treated at 25 and 50 °C also showed a decrease in *BS* values as compared with those corresponding to 10 °C. Therefore, the application of high HHP of 600 MPa, adopted as a pre-processing instrument in combination with heating processes, could reduce the thermo-hardening of heat-induced CF gel remarkably, leading to the development of new chickpea-based products that possess desirable handling properties and sensory attributes. Conversely, pressurization at the lowest level may be a desirable feature for consumers who prefer a firm, solid-like structure. At constant 400 MPa the *BS* values decreased significantly with increasing *T* from 10 to 50 °C, although an unexpected high *BS* value was obtained for samples HHP-treated at 400 MPa and 25 °C. 

Gel strength is an indirect measure of the nature of a viscoelastic material [40]. It also indicates the overall consistency of the formed gel that includes mechanical rigidity or hardness, cohesiveness or internal binding, and elasticity or springiness. Figure 5 shows the slope of CF gels as a function of pressure and *T*. The lowest brittleness is associated with the highest levels of these two factors. In turn, Figure 6 shows the *Coh* values from TPA tests obtained for the heat-induced CF gels after pressurization. It can be seen that pressure did not have a significant effect on this textural property, and the highest *Coh* value corresponded to unpressurized heat-induced CF gel. The effect of treatment *T* on the *Coh* of the gels was dependent on the pressure applied. At 200 and 600 MPa, there was a decrease in *Coh* when *T* was increased from 10 to 25 °C, but at the highest pressure *Coh* increased again when *T* was increased to 50 °C. Conversely, after pressurization at 400 MPa, *Coh* increased when *T* was increased from 10 to 25 °C, but scarcely changed at 50 °C. According to Katopo *et al*. [16], starch pressurized in powder form displays a lower gelatinization temperature and enthalpy change than native starch, indicating damage and loss of molecular order and crystallinity resulting from pressurization at 690 MPa.

Cohesiveness is a direct function of the work needed to overcome the internal bonding of the gel network [41]. Thus, when the gel network shows a higher degree of gelatinization, this means that the internal bonds of the gel structure are broken to a greater extent. Such a gel recovers less during the imitative second bite. However, although unpressurized CF gels had higher resistance to deformation, the impact of pressure and *T* on the textural properties derived from TPA tests was more ambiguous. Results would appear to indicate that the bloom strength test was a more adequate and suitable method for comparison of rigidity in the heat-induced CF gels after pressurization of the flour in powder form and, therefore, without water.

### 3.3. Microstructure Examination

#### 3.3.1. CF Gel Heat-Induced from HHP-Treated CF Slurry

**Figure 7:**
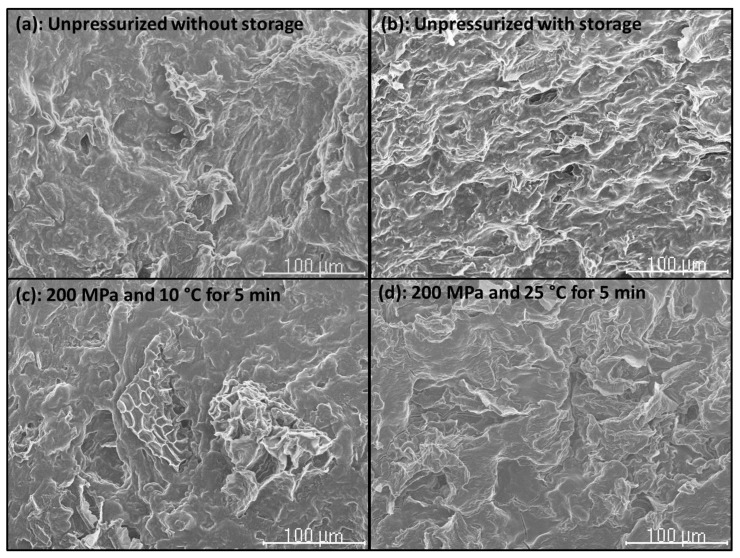
Scanning electron micrographs of unpressurized and pressurized heat-induced CF gels at (300×) magnification: (**a**) CF gel unpressurized without storage, (**b**) CF gel unpressurized with storage, (**c**) Heat-induced CF gel after pressurization at 200 MPa and 10 °C for 5 min, (**d**) Heat-induced CF gel after pressurization at 200 MPa and 25 °C for 5 min.

**Figure 8:**
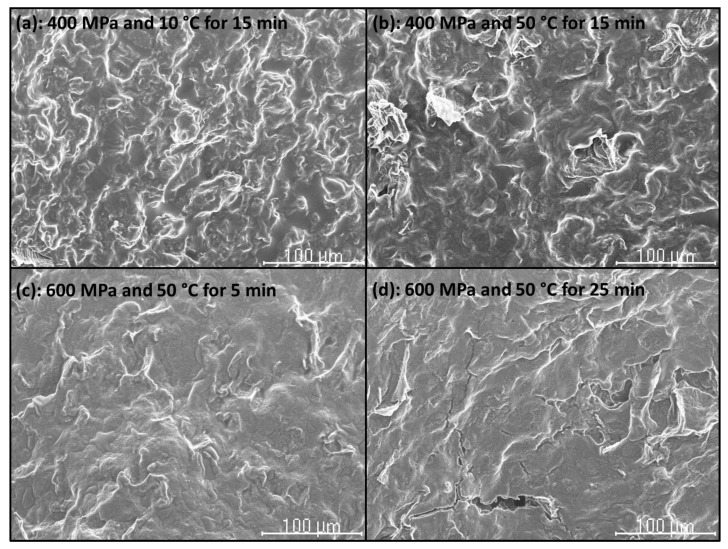
Scanning electron micrographs of pressurized heat-induced CF gels at (300×) magnification: (**a**) Heat-induced CF gel after pressurization at 400 MPa and 10 °C for 15 min, (**b**) Heat-induced CF gel after pressurization at 400 MPa and 50 °C for 15 min, (**c**) Heat-induced CF gel after pressurization at 600 MPa and 50 °C for 5 min, (**d**) Heat-induced CF gel after pressurization at 600 MPa and 50 °C for 5 min.

Figure 7 and Figure 8 show SEM micrographs of CF gels heat-induced from unpressurized and HHP-treated CF slurries under different combinations of pressure, *T*, and treatment *t*. Gels form entangled amylose solutions on cooling and occur as a result of a phase separation that produces a three-dimensional polymer network [2]. A gel was thus formed in all cases. During *T*-induced gelatinization, owing to the ingress of hot water, the hydrogen bonds between starch molecules are broken and hydrogen bonds with water are formed instead. Water is able to penetrate the granules further, and irreversible swelling takes place. Upon further heating, the granules begin to break down and amylopectin solubilization increases [38]. Figure 7a,b would appear to indicate that single *T*-induced gelatinization of unpressurized CF slurry without and with storage matches this behavior.

A previous study showed that weaker CF pastes are formed in the subsequent heating process following pressurization, because there is an increase in the amount of starch pre-gelatinized by pressure and the pastes are formed solely by melting of the crystallites that still remain [5]. In the present study, it is worth remembering that all the HHP-treated CF slurries were subjected to subsequent thermal heating, and micrographs of pressurized CF slurries were taken after additional *T*-induced gelatinization. Undoubtedly, this could mask the distinctive effect of the HHP treatment on the visual appearance and the HHP-induced starch gelatinization of the CF gels. A study by Vallons and Arendt [29] showed that, although preservation of granular structure was observed for buckwheat starch after treatment with 600 MPa, treatment with 75 °C resulted in complete disintegration of the buckwheat granules. In the present study there was no obvious difference between the samples subjected to 200 MPa at 10 or 25 °C for 5 min (Figure 7c,d) and without pressure treatment before isothermal heating, as isothermal heating at 75 °C for 15 min caused the loss of integrity of most granules.

Some cell walls can be distinguished in the CF slurries treated with 200 MPa at 10 °C for 5 min (Figure 7c). As mentioned above, the lowest pressure, *T*, and treatment *t* could cause an increase in the linearity of the cell walls and volumes of particles owing to the permeability of the cell walls [25]. Similarly, no differences were visible between untreated sorghum flour slurries and sorghum flour slurries treated with 200 MPa or 300 MPa at 20 °C for 10 min [42].

However, the pressure intensity, *T*, and treatment *t* significantly influenced the gelatinization of starch in the pressurized CF slurries (Figure 8). At higher pressure, 400 MPa at 10 or 50 °C for 15 min (Figure 8a,b), the samples became more solid. During gelatinization, the consistency of a starch-containing suspension will increase owing to leaching of amylose and swelling of the granules [43]. Therefore it can be assumed that the increased consistency of the CF slurry HHP-treated at 400 MPa and 10 °C for 15 min, as indicated by the high *G*’ and low tan *δ* values in the frequency sweep tests (Table 2), was due to the HHP-induced gelatinization of starch.

A higher *G*’_0_ indicated a higher degree of gelatinization of the starch caused by the HHP-treatment prior to pasting [44], and the results showed a sigmoidal-shaped relationship between degree of gelatinization and pressure applied, as previously reported by Stute *et al*. [10], Douzals *et al*. [13], Oh *et al*. [7,26], and Vallons and Arendt [9,29]. Similarly, Vallons *et al*. [42] showed that in sorghum batters treatment with pressures ≥400 MPa resulted in microstructural changes. The authors just cited found that the starch granules became swollen and deformed; nevertheless, even after treatment with 600 MPa at 20 °C for 10 min their granular structure remained intact.

However, in this study higher treatment *T*s and *t*s were applied. In particular, pressurization of CF slurry at the highest levels of the three factors (600 MPa and 50 °C for 25 min) clearly affected its appearance (Figure 8d), and the structure appears to be coated by a glue-like substance. The micrograph would seem to indicate that breakdown of granules and some splitting of macromolecules could be assumed at 600 MPa and higher *T*s and *t*s. In these cases, retrogradation probably occurred outside the granules, explaining why the viscoelastic parameter values obtained for the CF slurries HHP-treated at 600 MPa and 50 °C were higher for 25 min than for 15 min (Table 2). Other publications showed the presence of a residual crystalline order after HHP treatment, referred to as “rapid retrogradation” occurring even during or immediately after pressurization [4,10], and the greater the degree of gelatinization induced by the HHP treatment, the greater the extent of “rapid retrogradation”.

However, a number of factors have been shown to influence and alter the *G*’ of starch pastes and gels [45]. Tsai *et al*. [46] showed that the *G*’ of waxy maize starch increased after cross-linking even though the swelling power decreased. This implies that the *G*’ of starch pastes is also affected by the rigidity of gelatinized starch granules. Debet and Gidley [47] hypothesized that granule remnant or “ghost” formation is attributable to cross-linking of amylose and/or long amylopectin chains within swollen granules, most likely involving double helices. They stated that “ghost” formation involves a competition between swelling and cross-linking within the swollen granules. It has also been hypothesized that the protein contributes to the rigidity of swollen, gelatinized starch [45]. The authors just cited indicated that starch granule-associated proteins influence endosperm texture, and the gelatinization and pasting properties of starch. In addition to starch, CF slurries also contain a relatively large amount of protein [1,5]. We thought that the CF slurries HHP-treated at 600 MPa and 50 °C for 25 min contained starch ghosts, as a sticky surface film or coat enveloping this slurry was also clearly perceptible to the eye after pressurization under these conditions. The surface regions of many granules contain proteins and lipids that provide potential for film formation [47]. It has been stated that HHP treatment changes the conformation and coagulation of proteins by opening the native structures, resulting in denaturation and aggregation; affects the melting properties of starches and the rearrangement of the polymorphic forms in lipids; inactivates microorganisms; and induces chemical changes at low temperatures [48]. It could be possible to denature the protein components of lentils completely by a combination of pressure and elevated temperature [12]. In turn, overlapping protein and starch peaks in the CF thermogram made it impossible to determine the enthalpy of starch gelatinization and the width of the starch gelatinization transition [49].

#### 3.3.2. HHP-Treated CF in Powder Form

**Figure 9:**
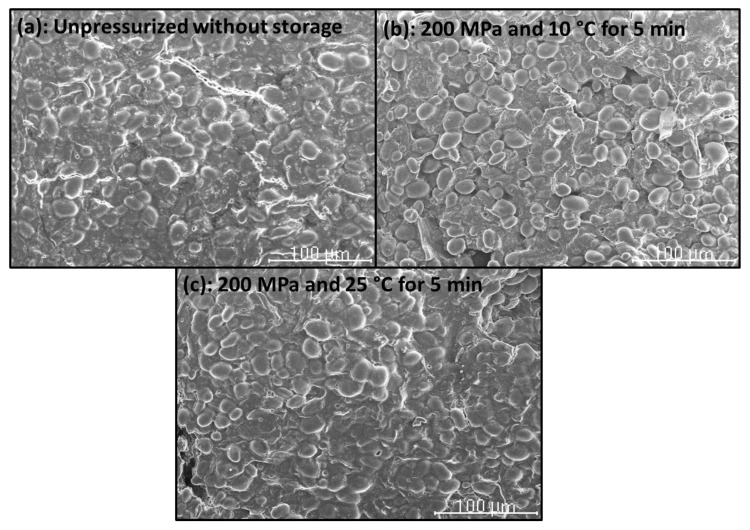
Scanning electron micrographs of unpressurized and pressurized CF in powder form at (300×) magnification: (**a**) Unpressurized CF without storage, (**b**) CF after pressurization at 200 MPa and 10 °C for 5 min, (**c**) CF after pressurization at 200 MPa and 25 °C for 5 min.

The structure of unpressurized and HHP-treated CF was also examined by SEM. Figure 9 shows the micrographs of native CF in powder form and CF in powder form pressure-treated at 200 MPa and either 10 or 25 °C for 5 min. The HHP-treated CF in powder form had not been subjected to successive heating when the micrographs were taken, and therefore the effect of HHP on the chickpea flour/starch can be distinguished more easily. Cotyledon cells of raw chickpeas showed that cell walls create a regular structure in which starch granules are enclosed [48]. According to the authors just cited, in raw chickpeas starch granules are the most representative storage components and appear to be embedded in a protein matrix. In unpressurized or native CF, the starch occurred as granules (Figure 9a). The starch granules of raw chickpeas tend to be elliptic and/or globular, smooth-surfaced, and range from 10 × 14 to 19 × 10 μm [50]. According to other authors, these untreated starch granules would exhibit characteristic granular shapes and birefringence patterns under polarized light [4,9,14]. In unpressurized CF, the starch granules exhibited a smooth surface with no evidence of fissures or pin holes, surrounded by well-defined protein bodies or fragments of protein matrix disrupted during milling, in agreement with the literature [51].

It can be seen that there is no observable difference for the samples without and with pressure treatment at 200 and 10 or 25 °C for 5 min (Figure 9b,c). This indicates that this low pressure may enhance the diffusion of water into the amorphous phase but is not high enough to destroy the crystalline structure [14]. There are no discernable changes in size, but the surface of the treated samples has become smoother. The smooth surface can also be explained by the water diffusion and granule swelling. Therefore, the SEM micrographs suggest preservation of granular structure after treatment with 200 MPa at 10 and 25 °C for the shortest treatment *t*. Vallons and Arendt [29] observed that the number of granules showing a “Maltese cross” decreased with increasing HHP or *T* above 300 MPa and 60 °C, respectively, indicating loss of crystallinity.

**Figure 10:**
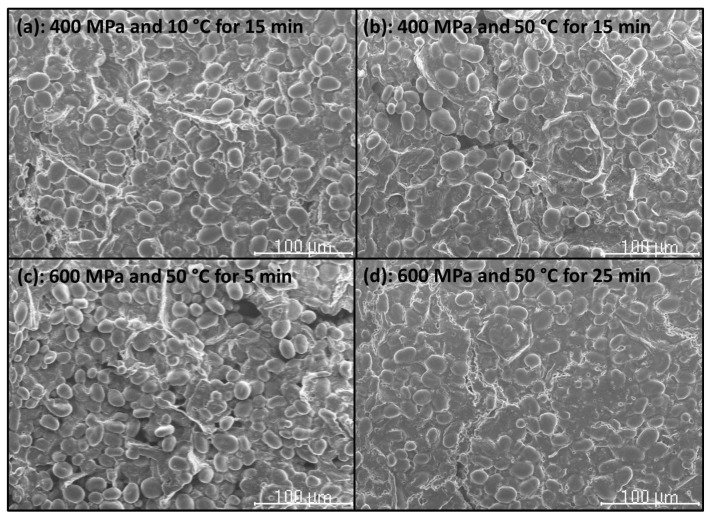
Scanning electron micrographs of pressurized CF in powder form at (300×) magnification: (**a**) CF after pressurization at 400 MPa and 10 °C for 15 min, (**b**) CF after pressurization at 400 MPa and 50 °C for 15 min, (**c**) CF after pressurization at 600 MPa and 50 °C for 5 min, (**d**) CF after pressurization at 600 MPa and 50 °C for 25 min.

However, in this study, after pressurization at 400 MPa and 10 or 50 °C for 15 min (Figure 10a,b), visible morphological changes were not found, and the integrity of the granules was maintained without fragmentation. The starch granules remained intact after the pressure treatment and no leaching of amylose was observed [8]. In comparison with heat gelatinization, however, most starches show limited swelling upon pressurization [7,10]. Lower swelling indices were observed for pressure-treated wheat, corn, and rice starch compared with heated starches [4,13]. According to Douzals *et al*. [13], limited granule expansion upon pressurization is the result of lower starch hydration at higher pressure. In addition, those authors stated that lower water binding at higher pressures leads to a lower release of amylose from the granules.

Stolt *et al*. [8] also reported no leaching of amylose during pressurization of a barley starch suspension at 550 MPa. As restricted swelling and lower release of amylose stabilize the granular structure, pressure-treated starches exhibit better preservation of the granular structure [13]. It was postulated by Oh *et al*. [26] that during pressure treatment amylose may form thermodynamically favorable complexes with displaced amylopectin molecules instead of leaching into the aqueous phase, resulting in limited swelling and more rigid granule remnants.

On the other hand, changes in the shape and surface of chickpea starch granules induced by treatment with HHP at 600 MPa and 50 °C for 5 min were not clearly visible. A study by Vallons and Arendt [29] showed that, although preservation of granular structure was observed for buckwheat starch after treatment with 600 MPa, treatment with 75 °C resulted in complete disintegration of the buckwheat granules. Finally, in this study, after pressurization at the highest levels of the three factors (600 MPa and 50 °C for 25 min), there was more surface cracking and some granules fused with each other (Figure 10d), although a good granule preservation can still be observed. We thought that the structural changes of pressurized CF in powder form were less drastic than those of HHP-treated CF slurries as a consequence of the limited presence of water. However, these differences were masked by the combination of both pressure- and temperature-induced gelatinization. More research will need to be conducted to study the effect of pressure, *T*, and treatment *t* on the microstructural features of HHP-treated CF slurries prior to heating.

## 4. Conclusions

The possibility of exploring differences in the degree of gelatinization induced by HHP treatments exists in the form of oscillatory rheometry and instrumental texture. The effect of pressure, *T*, and treatment *t* was detectable after either further heating or cooling, being more significant when the HHP treatments were applied to the CF slurries than to the CF in powder form. Although the effect of pressure was dependent on either *T* or treatment *t*, the impact of pressure on CF gelatinization was much more significant. The HHP-treated CF slurry exhibited weak-gel behavior with *G*’ > *G*” upon heating and cooling. The viscoelasticity and mechanical strength of a thermally-induced CF gel mostly decreased as a function of pressure applied in proportion to the extent of HHP-induced gelatinization of starch. On the other hand, as usual, the retrogradation of pressure-induced CF slurries prior to *T*-induced gelatinization increased the gel strength. The bloom strength test was found more suitable for studying these three effects all together on the texture of the CF gels heat-induced from pressurized powders. Increasing *T* also increased the degree of gelatinization of slurries pressurized at 400 and 600 MPa, and accordingly the CF gels induced subsequently had inferior mechanical strength. The elasticity of CF slurries treated at 200 and 400 MPa and 10, 25, and 50 °C decreased with increasing holding *t* in proportion to the extent of HHP-induced gelatinization. However, as a consequence of the significance of the interactions between the effects studied, many of the observations made cannot be explained so far. Consequently, more research needs to be done. Most of the results obtained for CF slurries pre-treated with 600 MPa at 50 °C for 25 min could be explained by various mechanisms and probably one mechanism is not the sole origin for the altered rheological properties. The results show, on the other hand, that, within CF, the influence of protein and starch-protein mixtures on the rheological properties and mechanical strength cannot be excluded. The information gathered from rheological and textural measurements needs to be related to other analyses to characterize the HHP-induced gelatinization and/or denaturation of starch and proteins of CF slurry. Overall, the results showed that the characteristics of CF starch granules can be modified by HHP pre-treatments to obtain CF-based products with easier handling properties.
